# Neural tracking of natural speech: an effective marker for post-stroke aphasia

**DOI:** 10.1093/braincomms/fcaf095

**Published:** 2025-03-10

**Authors:** Pieter De Clercq, Jill Kries, Ramtin Mehraram, Jonas Vanthornhout, Tom Francart, Maaike Vandermosten

**Affiliations:** Experimental Oto-Rhino-Laryngology, Department of Neurosciences, Leuven Brain Institute, KU Leuven, 3000 Leuven, Belgium; Experimental Oto-Rhino-Laryngology, Department of Neurosciences, Leuven Brain Institute, KU Leuven, 3000 Leuven, Belgium; Experimental Oto-Rhino-Laryngology, Department of Neurosciences, Leuven Brain Institute, KU Leuven, 3000 Leuven, Belgium; Experimental Oto-Rhino-Laryngology, Department of Neurosciences, Leuven Brain Institute, KU Leuven, 3000 Leuven, Belgium; Experimental Oto-Rhino-Laryngology, Department of Neurosciences, Leuven Brain Institute, KU Leuven, 3000 Leuven, Belgium; Experimental Oto-Rhino-Laryngology, Department of Neurosciences, Leuven Brain Institute, KU Leuven, 3000 Leuven, Belgium

**Keywords:** aphasia, natural speech processing, neural envelope tracking, diagnostics

## Abstract

After a stroke, approximately one-third of patients suffer from aphasia, a language disorder that impairs communication ability. Behavioural tests are the current standard to detect aphasia, but they are time-consuming, have limited ecological validity and require active patient cooperation. To address these limitations, we tested the potential of EEG-based neural envelope tracking of natural speech. The technique investigates the neural response to the temporal envelope of speech, which is critical for speech understanding by encompassing cues for detecting and segmenting linguistic units (e.g. phrases, words and phonemes). We recorded EEG from 26 individuals with aphasia in the chronic phase after stroke (>6 months post-stroke) and 22 healthy controls while they listened to a 25-min story. We quantified neural envelope tracking in a broadband frequency range as well as in the delta, theta, alpha, beta and gamma frequency bands using mutual information analyses. Besides group differences in neural tracking measures, we also tested its suitability for detecting aphasia at the individual level using a support vector machine classifier. We further investigated the reliability of neural envelope tracking and the required recording length for accurate aphasia detection. Our results showed that individuals with aphasia had decreased encoding of the envelope compared to controls in the broad, delta, theta and gamma bands, which aligns with the assumed role of these bands in auditory and linguistic processing of speech. Neural tracking in these frequency bands effectively captured aphasia at the individual level, with a classification accuracy of 83.33% and an area under the curve of 89.16%. Moreover, we demonstrated that high-accuracy detection of aphasia can be achieved in a time-efficient (5–7 min) and highly reliable manner (split-half reliability correlations between *R* = 0.61 and *R* = 0.96 across frequency bands). In this study, we identified specific neural response characteristics to natural speech that are impaired in individuals with aphasia, holding promise as a potential biomarker for the condition. Furthermore, we demonstrate that the neural tracking technique can discriminate aphasia from healthy controls at the individual level with high accuracy, and in a reliable and time-efficient manner. Our findings represent a significant advance towards more automated, objective and ecologically valid assessments of language impairments in aphasia.

## Teaser

The study examined brain responses to natural speech, i.e. neural tracking, in post-stroke aphasia. Individuals with aphasia exhibited reduced neural tracking of speech compared to healthy controls. Furthermore, neural tracking emerged as an accurate, sensitive, reliable and time-efficient marker for capturing aphasia at the individual level.

## Graphical Abstract

**Graphical Abstract:**
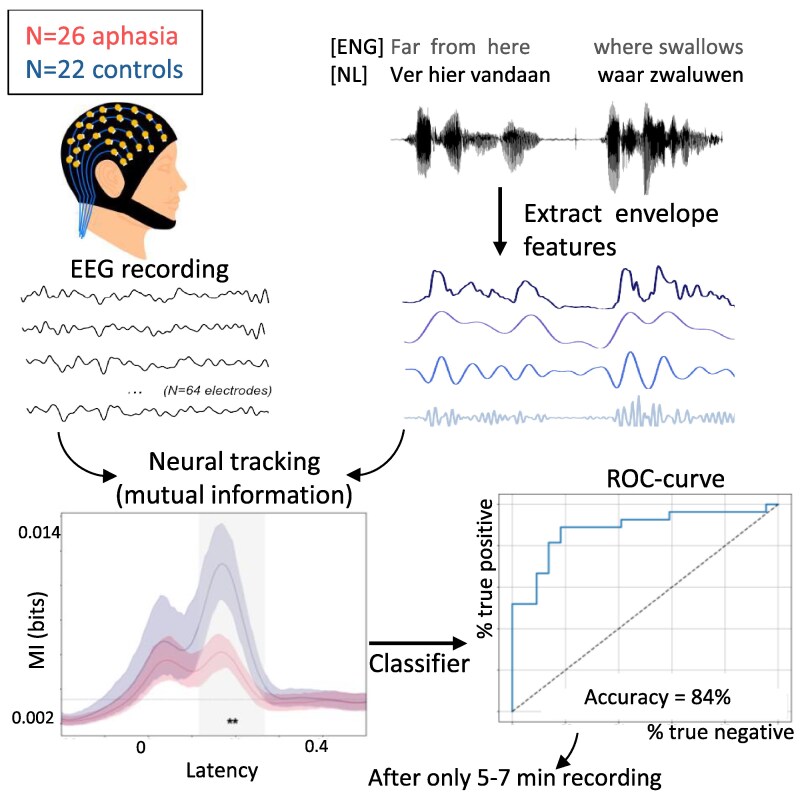

## Introduction

Aphasia is an acquired language disorder impairing communication ability and is principally caused by a stroke in the language-dominant left hemisphere. The current practice is to diagnose aphasia by means of standardized behavioural language tests. However, behavioural testing is time-consuming, requires active cooperation of the patient and can be biased by comorbid cognitive and motor impairments.^1^ Event-related potential (ERP) experiments have been conducted in aphasia, as EEG would be less biased by comorbid cognitive and motor problems. In response to language stimuli, individuals with aphasia (IWA) display altered ERP components,^2,3^ which have been argued to carry diagnostic value for aphasia.^4^ However, the reliability and sensitivity of ERPs to capture aphasia at the individual level are currently unknown. In addition, both behavioural tests and ERP paradigms do not reflect natural language as they rely on sounds, phonemes, words, or short sentences presented in isolation.^5^ Consequently, discrepancies exist between the test outcome and a patient's everyday-life language abilities.^6,7^

Recent advances in EEG research investigated the neural response to natural, running speech, which could open new perspectives to study natural speech processing in aphasia, hence increasing the ecologic validity. When we listen to speech, the brain tracks the temporal envelope, which consists of the slow-varying temporal modulations in the speech signal. These modulations contain essential cues for segmenting and identifying lexical units and prosody.^8^ In fact, research has shown that listeners can understand speech based on the temporal envelope only.^9^ Neural envelope tracking can be measured by applying encoding and decoding models to the data and is quantified by the model's ability to predict the EEG or decode the envelope. In a linear^10^ or a non-linear mutual information^11^ approach, outcomes can be visualized over time and space, revealing response properties similar to traditional ERP components.^12^ Neural envelope tracking is strongly related to speech understanding^13,14^ and can be leveraged to objectively quantify speech intelligibility.^15,16^

The neural response to the envelope in specific frequency bands reflects different hierarchical stages of speech processing.^17^ The delta band (0.5–4 Hz) is involved in processing higher-level linguistic structures of speech,^18,19^ while the theta band (4–8 Hz) tracks syllables and acoustic cues.^14,20^ These lower bands are believed to contribute most strongly to speech understanding. In higher frequencies, the alpha and beta bands are associated with attention and auditory–motor coupling,^21,22^ while gamma band is involved in encoding phonetic features.^23^

Previous studies have shown the potential of neural envelope tracking to capture language impairments. For individuals with primary progressive aphasia, a language disorder caused by a neurodegenerative disease, Dial *et al*.^24^ found increased theta band tracking, likely reflecting a compensation mechanism through increased reliance on acoustic cues. For individuals with dyslexia, characterized by phonological processing difficulties, decreased tracking in delta, theta and beta/gamma (phoneme- and phonetic-level) bands has been reported.^25-27^

The present study investigated whether neural envelope tracking using mutual information (MI) analyses can differentiate IWA in the chronic phase after stroke (≥6 months post-stroke) from neurologically healthy, age-matched controls. We described both groups’ responses to the speech envelope at broadband and in specific frequencies ranging from delta to gamma band. We further assessed whether aphasia from healthy aging can be discriminated at the individual level using a support vector machine (SVM) classifying held-out participants as healthy or aphasic. Finally, the reliability and minimal required recording length of the technique were evaluated.

## Materials and methods

### Participants

Our sample comprised 26 IWA (seven female participants, mean age = 72 years, SD = 15 years old) in the chronic phase (≥6 months) after stroke and 22 neurologically healthy controls (seven female participants, mean age = 72 years, SD = 7 years old). There was no significant age difference between groups (unpaired Wilcoxon rank-sum test: *W* = 321.5.5, *P* = 0.47). IWA were recruited at the stroke unit of the University Hospital Leuven [all patients failed the Dutch Language Screening Test (LAST)^28^ administered at the stroke unit with a score below cut-off for aphasia] and via speech-language pathologists. Healthy controls were recruited, making sure they matched the age of IWA at the group level. The inclusion criteria for IWA were: (i) a left-hemispheric or bilateral stroke; (ii) a diagnosis of aphasia in the acute stage after stroke using behavioural language tests; and (iii) no formal diagnosis of a psychiatric or neurodegenerative disorder. A detailed overview including demographic information (age, sex, time since stroke, speech-language therapy, performance on diagnostic tests for aphasia at time of participation) and lesion information (stroke type, affected blood vessel, lesion size and lesioned hemisphere) about the aphasia sample can be found in the Supplementary Table 1. For more information regarding recruitment strategy and diagnosis in the acute stage after stroke, we refer to Kries *et al*.^29^ The study was approved by the ethical committee UZ/KU Leuven (S60007), and all participants gave written consent before participation. Research was conducted in accordance with the principles embodied in the Declaration of Helsinki and in accordance with local statutory requirements.

Participants completed standardized clinical tests for aphasia at the time of participation (i.e. >6 months post-stroke) as described in detail in Kries *et al*.^29^ IWA scored significantly lower on the ‘Nederlandse Benoemtest’, i.e. Dutch Naming test,^30^ and the ScreeLing test^31,32^ compared to healthy controls (*W* = 55.5, *P* < 0.001; *W* = 101, *P* = 0.001, respectively). The ScreeLing test is a comprehensive aphasia test battery testing phonological, semantic and syntactic language processes, consisting of both receptive and expressive test items. Although eight IWA did not score below the cut-off threshold for aphasia on either the ScreeLing or the naming tasks, they were included in this study since they still attended speech-language therapy sessions at the time of participation and had extended documentation of language deficits in the acute stage after stroke.^29^

**Figure 1:**
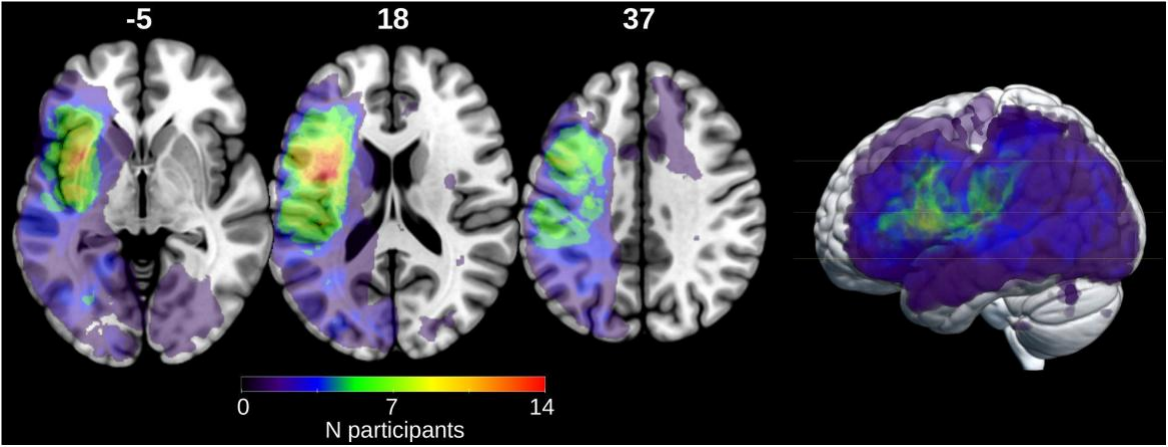
**Lesion overlay image (max overlap = 14) of the sample (*N* = 25 participants).** Axial slices with corresponding *Z* coordinates are shown in neurological orientation.

A lesion overlap map of our aphasia sample is provided in Fig. 1. The segmentation of lesioned stroke tissue was performed on T_2_-weighted fluid-attenuated inversion recovery images obtained during the acute stage at the University Hospital of Leuven for 15 participants, and during the chronic stage (intended for a future study) for 10 participants. One participant (sub-038, see Supplementary Table 1) lacked accessible brain scan data, but was included in the study as the participant had a confirmed diagnosis of aphasia and scored below the aphasia cut-off threshold on both diagnostic tests (ScreeLing and Dutch Naming tests) at the time of participation. For 12 participants who had previously participated in a prior study,^33^ we utilized the previously established segmentation maps. The remaining 13 scans were manually segmented, guided by the information documented in their respective medical files.

### EEG experiment

The EEG measurements took place in a soundproof, electromagnetically shielded booth using a 64-channel BioSemi ActiveTwo system (Amsterdam, the Netherlands) at a sampling frequency of 8192 Hz. Participants were instructed to listen to a 25-min-long story, *De Wilde Zwanen*, written by Christian Andersen and narrated by a female Flemish-native speaker, presented in silence while their EEG was recorded. The story was cut into five parts with an average duration of 4.84 min. After each story part, participants answered content questions about the preceding part, introduced to make the participant follow the content attentively. IWA scored significantly lower on these questions, with data and statistics presented in the Supplementary Fig. 1. However, as these questions were not validated, this result is not part of the main analysis and should be seen as descriptive information. As one participant fell asleep during two story parts, we left these parts out of the analysis for that participant (sub-019, see Supplementary Table 1). The story was presented bilaterally through ER-3A insert earphones (Etymotic Research Inc, IL, USA) using the software platform APEX.^33^

We determined a subject-dependent intensity level at which the story was presented based on the thresholds of the pitch tone audiometry (PTA). We defined hearing thresholds for octave frequencies between 0.25 and 4 kHz. For normal hearing participants, the story was presented at 60 dBA. For hearing impaired participants, defined as participants that have a hearing threshold > 25 dB hearing loss on frequencies below 4 kHz, the volume was augmented with half of the pure tone average of the individual thresholds at 0.25, 0.5 and 1 kHz for both ears individually. This procedure was adapted from Jansen *et al*.^34^ To check whether age-related hearing loss differed between both groups, we calculated the Fletcher index, i.e. average of PTA thresholds at 0.5, 1 and 2 kHz. Hearing levels did not differ between groups (Fletcher index averaged across the right and left ears: *W* = 308, *P* = 0.66).

### Signal processing

#### Envelope extraction

We used a gammatone filter bank^35^ to extract the envelope. We used 28 channels spaced by one equivalent rectangular bandwidth and centre frequencies from 50 Hz until 5000 Hz. The envelopes were extracted from each sub-band by taking the absolute value of each sample and raising it to the power of 0.6. The resulting 28 sub-band envelopes were averaged to obtain a single envelope. Next, the envelope was downsampled to 512 Hz to decrease processing time using an anti-aliasing filter (with the built-in MATLAB resample function). The envelope was then filtered in frequency ranges of interest. These include delta (0.5–4 Hz), theta (4–8 Hz), alpha (8–12 Hz), beta (12–30 Hz), low-gamma (30–49 Hz) and a broad (0.5–49 Hz, including all individual frequency ranges) bands. We used high- and lowpass filters, with a stopband of 10% below the highpass and 10% above the lowpass frequency, with a maximal ripple of 0.25 dB in the passband and an attenuation of at least 10 dB at the stopband frequency. A Least Squares filter was used, and we compensated for the group delay. The order of the filter was chosen in an incremental approach, with order increasing until the ripple and attenuation-criteria were met. This led to an order of 2000 for all filters < 30 Hz (both low- and highpass), and an order of 500 for filters ≥ 30 Hz. The impulse and frequency response of all filters are provided in the Supplementary Fig. 2. After filtering, the envelope was *Z*-score-normalized and further downsampled to 128 Hz using an anti-aliasing filter.

#### EEG data pre-processing

EEG data were pre-processed using the Automagic toolbox^36^ and custom MATLAB scripts (The MathWorks Inc., Natick, MA, USA, 2021). The EEG signals were first downsampled to 512 Hz to decrease processing time using an anti-aliasing filter. Line noise was removed using the ZapLine method,^37^ and EEG signals were cleaned from bad channels (i.e. defined based on correlations with neighbouring EEG channels using the RANSAC algorithm implemented in the PREP pipeline^38^) using spherical interpolation, with no difference between groups in the number of interpolated channels (average number of channels aphasia = 1.62, SD = 1.92; average number of channels controls = 1.43, SD = 1.31; Wilcoxon rank-sum test: *W* = 258, *P* = 0.73). Artefactual segments of data were replaced with a temporal interpolation using the artefact subspace reconstruction method.^39^ Next, an independent component analysis was applied to the data, and components classified as ‘brain’ or ‘other’ (i.e. mixed components), using the EEGLAB plugin ICLabel,^40^ with a probability higher than 50% were preserved (average number of removed components: 26, SD = 7; average number of retained components: 38, SD = 7). There was no significant difference in the number of removed components between groups (average number of components aphasia = 27, SD = 6; average number of components controls = 24, SD = 8; Wilcoxon rank-sum test: *W* = 343, *P* = 0.15). The neural signals were projected back to the channel space, where the signals were common-average referenced. Subsequently, we filtered the EEG data in the same frequency bands using the same Least Squares filter as in the envelope extraction method. Next, *Z*-score normalization and further downsampling to 128 Hz were applied using an anti-aliasing filter.

### Neural envelope tracking

We investigated neural envelope tracking using the Gaussian copula MI analysis.^41^ In the Gaussian copula approach, all variables (the envelope and EEG channels) are first ranked on a scale from 0 to 1, obtaining the cumulative density function (CDF). By computing the inverse standard normal CDF, the data distributions of all variables are transformed to perfect standard Gaussians. Subsequently, the parametric Gaussian MI estimate can be applied to the data provided by:


(1) $$I(X;Y)=\frac{1}{2\ln2}\left[\frac{\left|\sum X\right|\left|\sum Y\right|}{\left|\sum XY\right|}\right]$$

where *I*(*X*;*Y*) equals the MI between *X* and *Y* (here, the EEG and the envelope), expressed in bits. |∑X| and |∑Y| are the determinants of the covariance matrices of *X* and *Y*, and |∑XY| is the determinant of the covariance matrix for the joint variable. To obtain temporal information on MI, we shifted the EEG as a function of the envelope over time (using an integration window −200–500 ms) and applied Eq. (1) at each sample. The result forms the temporal mutual information function (TMIF) and reflects how the brain processes speech over time.^11,42^ For an in-depth explanation of the Gaussian copula MI method, we refer to Ince *et al*.^41^ For a more practical explanation of the TMIF in the context of neural envelope tracking, and its added value over linear models, see De Clercq *et al*.^11^

We calculated the single-channel TMIF and the multivariate TMIF, analogue to a (linear) encoding and decoding model, respectively. The single-channel TMIF calculates the TMIF for each channel individually, providing both temporal (i.e. peak latency and peak magnitude) and spatial (i.e. topography) information on speech processing. Alternatively, the multivariate TMIF determines the multivariate relationship between multiple EEG channels combined and the speech envelope. This latter method is statistically more powerful as it takes interactions between EEG channels into account. However, it is restricted to temporal interpretations only. Prior to visualization, the time-resolved TMIFs were convolved with a Gaussian kernel of nine samples (SD = 2) to obtain smooth outcomes over time, a procedure often applied in linear TRF modelling as well (e.g. see Van Hirtum *et al*.^43^). For the multivariate TMIF, we used a channel selection including fronto-central and parieto-occipital channels that contribute to speech processing.^44^ Our channel selection is visualized in the Supplementary Fig. 3.

#### Permutation testing

Neural tracking (MI in bits, in this case) is a relative metric and should be compared to a null-distribution to quantify the meaningfulness of the derived values.^11^ We created stationary noise that matched the spectrum of the envelope per frequency band individually. Next, we calculated the MI between the noise envelope and the EEG per participant and repeated this process 1000 times. The significance level was then determined as the 95th percentile of permutations per participant (resulting in a single significance level per participant).

No significant differences were found in the significance level between IWA and controls for any frequency band, as determined by Wilcoxon rank-sum tests (see Supplementary Fig. 4). As no significant differences in the significance level were found between groups, we used a single significance level (i.e. the 95th percentile of permutations across all participants) to interpret the multivariate TMIFs in the ‘Results’ section.

### Statistical analysis

#### Group comparisons

We compared neural envelope tracking for IWA with the control group for broadband as well as for delta, theta, alpha, beta and gamma frequency ranges. For the single-channel TMIF, we performed non-parametric spatio-temporal cluster-based permutation tests,^45^ indicating clusters in the TMIF over time and space with the largest group difference at threshold *P* < 0.05. For the multivariate TMIF, we performed non-parametric temporal cluster-based permutation tests,^45^ indicating clusters of samples with the largest group difference at threshold *P* < 0.05.

#### Support vector machine classification

We investigated whether EEG-based envelope tracking outcomes can be used for detecting aphasia. To this end, we used a SVM to classify held-out participants as control or aphasic using the Scikit-Learn (v. 0.24.2) library in Python.^46^ The multivariate TMIFs for all five individual frequency bands (delta to gamma) were used as input to the model. Additionally, we added age of the participant, as it influences neural envelope tracking.^47^ We chose a radial basis function kernel SVM and performed a nested cross-validation approach. In the inner cross-validation, the C-hyperparameter and pruning (i.e. length of the TMIFs) were optimized (accuracy-based) and tested in a validation set using 5-fold cross-validation. The trained model was then tested on the test set, for which we used a leave-one-subject-out cross-validation approach.

The performance of the SVM classifier was evaluated by computing the receiver operating characteristic (ROC) curve and calculating the area under the curve (AUC). We further reported the overall accuracy, the F1-score, the sensitivity and the specificity of the classifier.

#### Feature contribution

To obtain a proxy for the relevant contribution of each frequency band, we applied the SHAP (Shapley Additive exPlanations) analysis.^48^ SHAP values quantify the impact of individual features on the SVM's classification, with positive SHAP values denoting features pushing predictions towards the positive class (aphasia), negative SHAP values pushing predictions towards the negative class (healthy control), and values around 0 reflecting a limited feature impact.

### Recording time

#### Classification

From a clinical perspective, we were interested in how much data the neural envelope tracking technique requires to detect aphasia accurately and obtain stable, reliable results. We iteratively cropped the EEG recording and the envelope in steps of 2 min (using the first 1 min, first 3 min, 5, 7 … up to the entire 25 min of recording time) and calculated the TMIF per frequency band per time duration. Next, we investigated the amount of minutes required for the SVM to reach its classification potential. As described above, we trained and tested our SVM per time duration in the same fashion as the entire duration. Performance (AUC, accuracy, F1-score) was plotted as a function of recording time. We determined the knee point, i.e. the point at which the performance benefit starts to saturate, using the ‘kneed’ Python package.^49^ The knee point of this curve reflects the point at which the increase in model performance may no longer be worth the corresponding effort.

#### Within-subjects stability

Second, we investigated the data required to obtain stable, reliable results. We determined the within- and between-subjects stability per time duration. For the within-subjects stability, we individually correlated (intraclass correlations) the TMIF per time duration (i.e. first minute, first 3, 5, …) with the TMIF of the entire recording per subject. This resulted in a single correlation coefficient for each participant, frequency band and time duration. Next, all correlations were plotted as a function of recording time, and we determined the knee point of the curve on the average across all frequency bands. As such, we gained insight into the amount of data required for a participant's TMIF to become stable (i.e. when there is not much change in an individual's TMIF).

#### Between-subjects stability

For the between-subjects stability, we calculated each participant's mean MI of the TMIF (integration window 0–400 ms) per time duration (1, 3, 5, … minutes) and the entire recording. This resulted in a single datapoint per participant, frequency band and time duration (i.e. mean MI for a certain duration length × mean MI entire duration). Subsequently, we calculated the correlation coefficient (intraclass correlation) between the mean MI for certain time duration and frequency band with the entire recording over participants on the group level, resulting in a single correlation coefficient per time duration and frequency band. We plotted the correlations as a function of recording time and determined the knee point of the curve on the average across all frequency bands. With this analysis, we investigated the amount of data required for a participant's relative (i.e. compared to other participants) strength of tracking to become stable (i.e. from which point on a participant's relative neural tracking compared to other participants is no longer expected to change).

#### Split-half reliability

Finally, we report a traditional split-half reliability metric with non-overlapping parts of the recording. We split the EEG recording into two equal parts, i.e. the first 12.5 min and the second 12.5 min, and computed the TMIFs for each half and each frequency band individually. Next, we computed the mean MI value of the TMIF (0–400 ms) for the first and second halves of the recording per participant individually. Subsequently, we calculated one-way random effects intraclass correlation coefficients (ICC) between the first and second halves of the recording over participants on the group level for IWA and controls separately.

## Results

### Distinguishing individuals with aphasia from healthy controls

**Figure 2:**
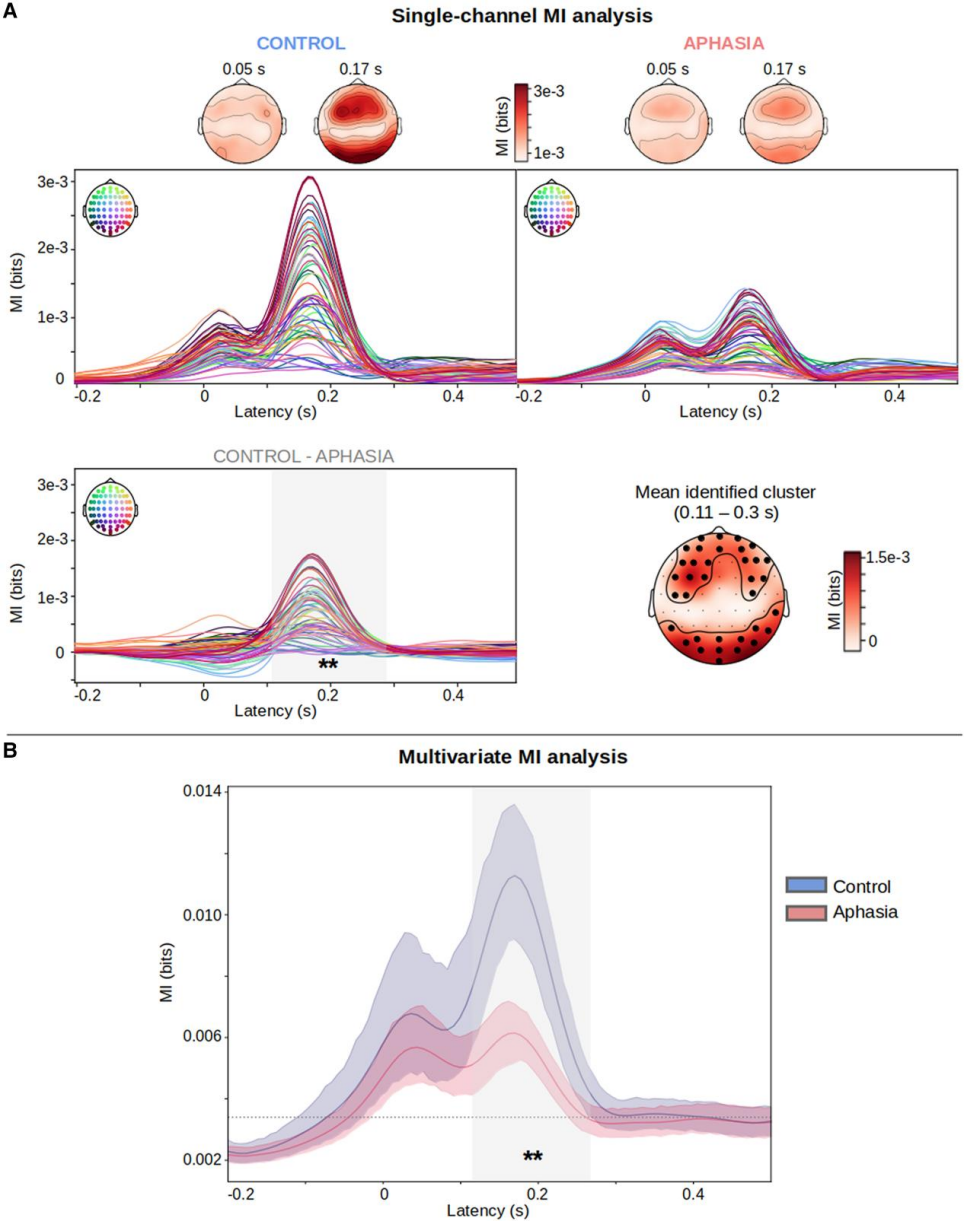
**Broadband analysis.** (**A**) The average single-channel TMIF for the control and the aphasia group separately, with topoplots at the first and second peaks (0.05 and 0.17 s). The spatio-temporal cluster-based permutation test investigated the difference between the control and aphasia group (control—aphasia) and identified a cluster (below threshold *P* < 0.05) with the largest group difference, centred around the second peak. Brain latencies belonging to the cluster are marked in a shaded grey area, the channels belonging to the cluster are indicated with a black dot on the topoplot. (**B**) The group average TMIF, for both groups separately. The shaded areas around the curves indicate the 95% confidence interval. The shaded grey area indicates the cluster with largest group difference (threshold *P* < 0.05), identified using a temporal cluster-based permutation test. **P < 0.01.

We investigated whether neural envelope tracking is altered in IWA compared to healthy controls. First, we studied the effect in the broadband frequency range (0.5–49 Hz). For the single-channel MI analysis, providing both temporal and spatial information, we found decreased neural envelope tracking for IWA compared to healthy controls (Fig. 2A). A spatio-temporal cluster-based permutation test identified a cluster comprising a large group of fronto-central, parietal and posterior channels (*N* = 42 channels) from 0.11 to 0.3 s (*P* = 0.005), centred around the second peak. The multivariate MI analysis, which combines information from multiple channels, confirmed these results: a temporal cluster-based permutation test identified a cluster between 0.11 and 0.26 s in which IWA displayed a decreased response (*P* = 0.005) (Fig. 2B).

**Figure 3:**
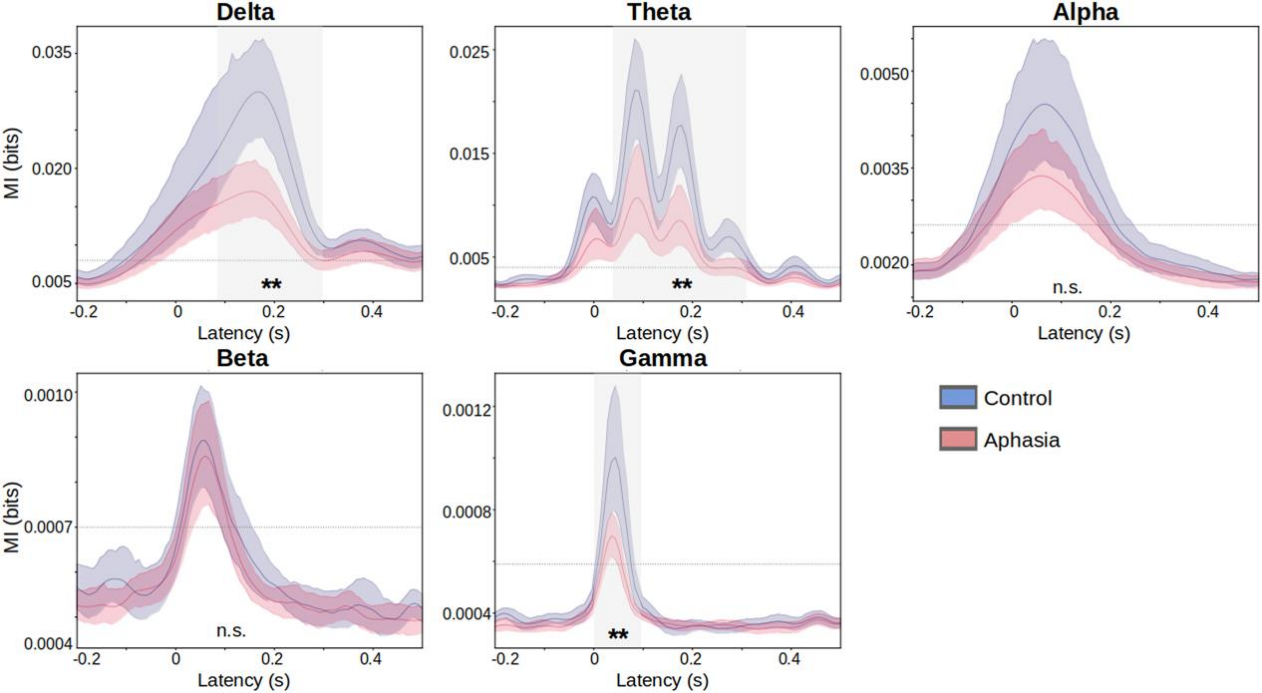
**Frequency-specific analysis.** Group average TMIF's visualized per frequency band, with coloured shaded areas indicating the 95% confidence interval. Shaded, grey areas indicate clusters with largest group difference (below threshold *P* < 0.05) identified using temporal cluster-based permutation tests. **P < 0.01.

We further investigated the neural response in narrow frequency bands. We focused on the multivariate TMIF as it is a statistically more robust method compared to the single-channel TMIF, and we used those features as input to our SVM classifier in the subsequent section. The single-channel TMIFs for all frequency bands are provided in the Supplementary Figs 5–9. We generally observed decreased neural envelope tracking for IWA compared to healthy controls (Fig. 3). Temporal cluster-based permutation tests identified clusters below threshold *P* < 0.05 for delta (0.1–0.30 s, *P* = 0.003), theta (0.01–0.31 s, *P* = 0.001) and gamma (0.01–0.1 s, *P* = 0.007) bands. No clusters exceeding the *P* < 0.05 threshold were detected for the alpha and beta bands. These results are confirmed in the single-channel MI analysis, where spatio-temporal cluster-based permutation tests identified clusters for delta, theta and gamma bands for a large group of fronto-central, parietal and posterior channels (visualizations and statistics provided in the Supplementary Figs 5–9).

### Support vector machine classification

**Figure 4:**
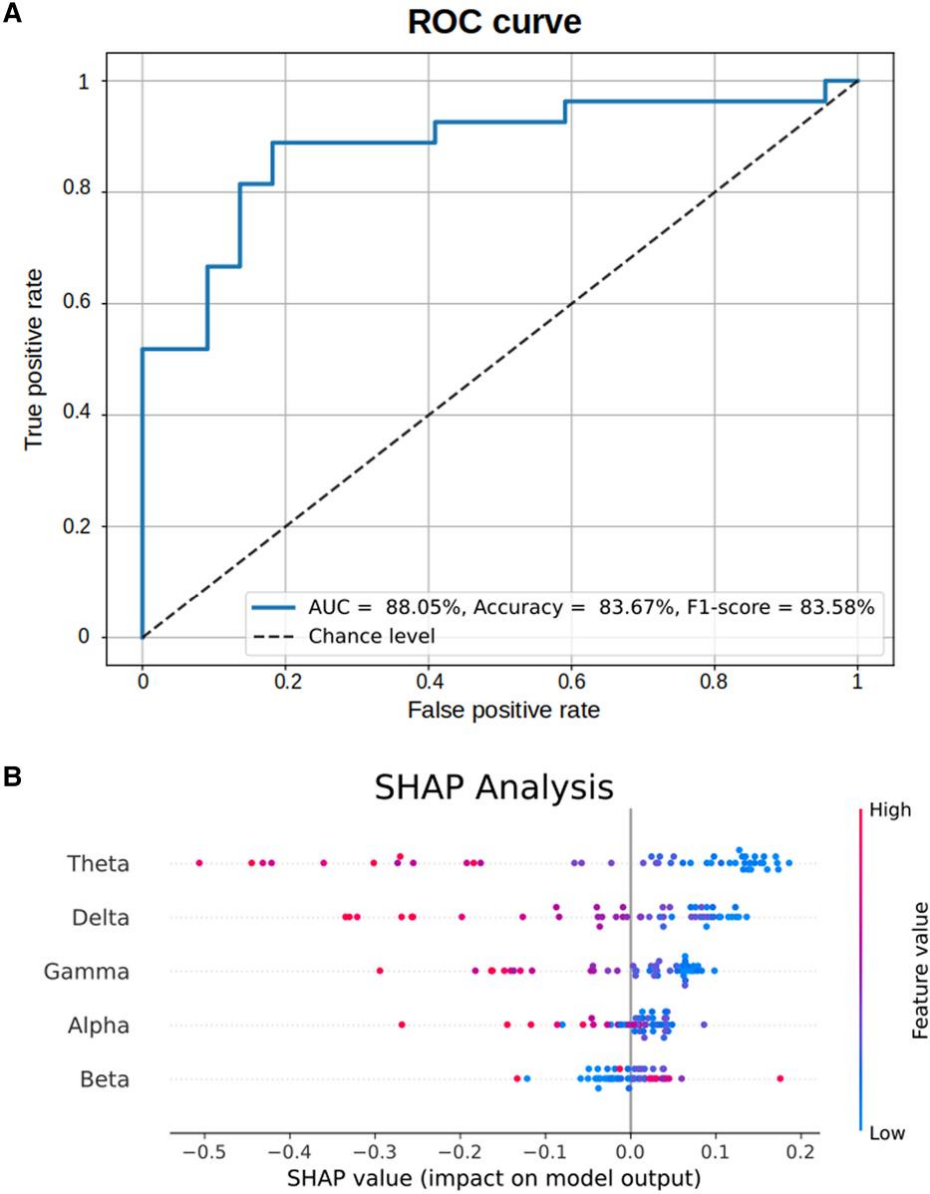
**Result of the SVM classifier.** (**A**) Receiver operating characteristic curve (ROC), with performance scores [AUC (=area under the curve, accuracy, F1-score]. (**B**) SHAP analysis results.

Next, we investigated whether we could detect aphasia based on neural envelope tracking measures in the individual frequency bands. To this end, we used an SVM to classify participants as belonging to the aphasia or the healthy control group via leave-one-out cross-validation. We used the TMIFs in our five frequency bands of interest and age as input features to the model. The SVM successfully classified participants belonging to either group with an accuracy of 83.33%, an F1-score of 83.25% and an AUC of 89.16%. The SVM had a sensitivity of 88.46% and a specificity of 77.27% for aphasia. Figure 4A displays the ROC curve.

To obtain a measure of relative frequency band contribution, we applied the SHAP analysis, which investigates the impact of individual feature values on SVM predictions. This result is visualized in Fig. 4B, where features are ranked according to their impact (top = most impactful feature). Positive SHAP values on the *x*-axis indicate predictions being pushed towards aphasia prediction (e.g. this is the case for low theta tracking), and negative SHAP values indicate predictions being pushed towards healthy control prediction (e.g. this is the case for high theta tracking). The SHAP analysis identified theta as the most impactful feature, followed by delta, gamma, alpha and beta.

### Recording length

**Figure 5:**
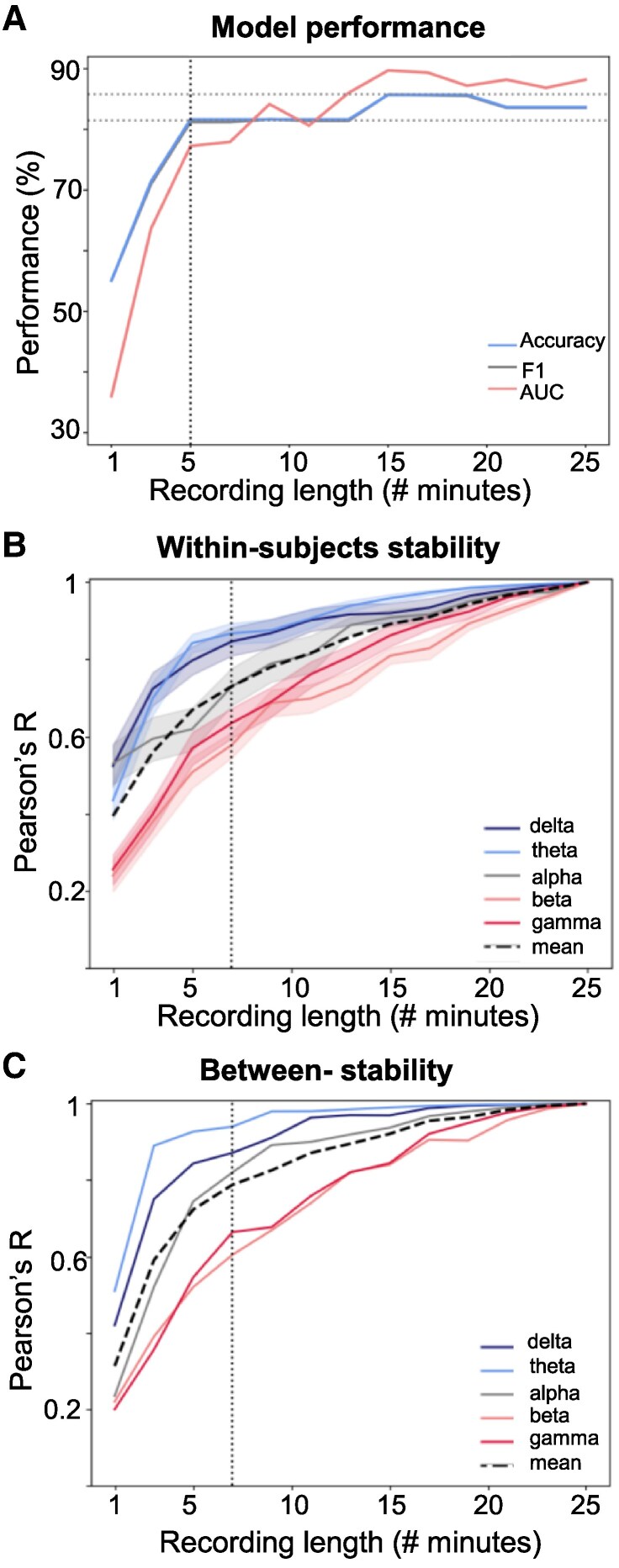
**Recording length.** (**A**) Performance [accuracy, F1-score, AUC (=area under the curve)] of the SVM classifier plotted as a function of time. (**B**) Within-subjects stability for all five frequency bands and the average across frequency bands (black line). Shaded areas indicate the standard error. (**C**) Between-subjects stability for all five frequency bands and the average across frequency bands (black line). The knee point of all panels is indicated with a vertical dotted line (based on the average for **B** and **C**).

We further investigated how much data the neural envelope tracking technique requires for robust and stable results (Fig. 5). With only 1 min of recording time, the SVM achieved a classification accuracy close to chance level (55%). Yet, the SVM reached an accuracy of 81.25% at the 5-min mark, which was identified as the knee point of the curve (see Fig. 4A). From 7 min on, the SVM consistently achieved an accuracy equal to the full recording length (83.33%), with peaks at 85.42%. Regarding the AUC, performance exceeded 80% from 9 min on. The F1-score mostly overlapped with the accuracy and never differed more than 0.88%. Figure 5A presents the SVM performance as a function of recording length.

The within-and between-subjects stability is plotted as a function of recording time in Fig. 5B and C. Highest within- and between-subjects correlations were observed for the low-frequency bands, namely delta and theta. Taking the average of all frequency bands, we identified the curve's knee point at 7 min of recording length (see black dotted lines). The within-subjects stability (Fig. 5B) had an average correlation of *R* = 0.73, and the between-subjects stability (Fig. 5C) had an average correlation of *R* = 0.79 at the knee point of the curve.

### Reliability of neural envelope tracking

Finally, we calculated the split-half reliability of neural envelope tracking. Table 1 provides the correlations and statistics. We generally found higher correlations in the lower frequency bands (delta and theta). Correlations were comparable between IWA and the control group; the 95% confidence intervals overlapped for each frequency band.

**Table 1:** Split-half reliability

	Delta		Theta		Alpha		Beta		Gamma	
	IWA	C	IWA	C	IWA	C	IWA	C	IWA	C
ICC	0.82	0.91	0.96	0.92	0.69	0.78	0.63	0.63	0.61	0.73
CI	[0.65; 0.92]	[0.80; 0.96]	[0.91; 0.98]	[0.82; 0.97]	[0.42; 0.84]	[0.53; 0.90]	[0.33; 0.81]	[0.31; 0.83]	[0.32; 0.80]	[0.45; 0.88]
*P*-value	<0.001	<0.001	<0.001	<0.001	<0.001	<0.001	<0.001	<0.001	<0.001	<0.001

CI, 95% confidence interval; C, control group. Fisher *z*-test comparing controls—IWA. All *P*-values are corrected for multiple comparisons.

## Discussion

We conducted an in-depth study on neural envelope tracking of natural speech in post-stroke aphasia. We found that IWA display decreased neural envelope tracking compared to healthy controls at broadband frequency range. Frequency-specific analyses indicated that group differences are most prominent in the delta, theta, and gamma frequency ranges. Furthermore, the suitability of neural envelope tracking measures as a biomarker for post-stroke aphasia was demonstrated using an SVM classifier that yielded high accuracy (83.33%, AUC 89.16%). Finally, we showed that an assessment based on neural envelope tracking could be obtained in a time-efficient (5–7 min of EEG recording) and reliable manner.

### Individuals with aphasia display decreased neural envelope tracking

#### Broadband frequency analysis

Neural envelope tracking at broadband is decreased in IWA compared to healthy controls. The single-channel TMIF analysis revealed a cluster at neural response latencies centred around the second peak in the TMIF comprising a large number of electrodes (see Fig. 2A). The multivariate TMIF confirmed this result: a temporal cluster comprising brain latencies surrounding the second peak in the TMIF (Fig. 2B) was identified. Recent work suggests that the second peak is primarily related to speech understanding, while the first peak is likely more implicated in acoustically processing the signal.^50,51^ Therefore, it is not surprising that in IWA, where language understanding is impaired, the second peak in the TMIF is decreased compared to healthy controls.

The temporal envelope of speech is labelled as an acoustic speech representation in the neural tracking literature. While often ignored in aphasia research, acoustic speech processes (e.g. rise time processing) are frequently impaired in IWA as well, and are associated with phonemic impairments, suggesting a cascading effect wherein deficits in lower-level acoustic processing impact higher-order language functions.^29^ The presented results in this study confirm acoustic processing impairments in aphasia at the neural level. Nevertheless, envelope processing also contributes significantly to higher-order speech and language understanding, as neural envelope tracking is reduced for incomprehensible speech or syntactically incorrect stimuli for healthy young listeners.^14,19,52,53^ Furthermore, neural envelope tracking has also been found implicated in top-down predictive coding processes.^54,55^ Therefore, reduced neural envelope tracking likely reflects a speech processing deficit in aphasia, although the specific underlying functional role (i.e. acoustic and auditory or higher-order language processing) must be assessed in future work.

#### Frequency-specific analysis

We further investigated neural envelope tracking in narrow frequency bands. Our findings revealed decreased envelope tracking for IWA compared to healthy controls in the low-frequency bands (delta and theta, see Fig. 3A and B), which encode syllables and higher-level linguistic units, and significantly contribute to speech understanding.^19,52,56^ Atypical neural tracking of the low-frequency temporal envelope has been reported in several other clinical populations, including individuals with primary progressive aphasia^24^ and dyslexia.^25,27^

Fewer studies investigated the role of high-frequency neural envelope tracking. Our findings revealed no group differences in the alpha and beta bands. However, IWA displayed decreased gamma band tracking, involved in encoding phonetic features.^23,57^ The gamma band has been of particular interest in dyslexia research, with several studies reporting alterations in gamma band activity. However, most studies have focused on phase-locking and phase coherence in response to amplitude-modulated noise (for a review, see Lizarazu *et al*.^26^), while few have investigated gamma band neural envelope tracking during natural speech processing.^27^ To gain a better understanding of (low-) gamma band neural envelope tracking of natural speech, for which we have shown robust group differences and significant contributions to detect aphasia (as depicted in Fig. 4B), future research should aim further to investigate its implications for speech understanding and language impairments.

To further explore whether individual frequency bands indeed reflect different speech processes, we performed additional analyses where correlations were calculated between neural tracking in individual frequency bands. Results revealed low to moderate correlations between different frequency bands (see Supplementary Table 2). By contrast, broadband frequency displays high redundancy with the delta band (*R* = 0.79 for IWA, *R* = 0.92 for controls), which can be attributed to the fact that most power in the EEG and the envelope is concentrated in the lowest frequencies. This highlights the relevance of conducting frequency-specific analyses, which favoured good SVM classification results in our study.

#### Accurate, time-efficient and reliable individual analyses

We assessed the ability of the neural tracking technique to detect post-stroke aphasia using an SVM classifier with the TMIFs computed in the individual frequency bands as input to the model. The SVM robustly detected aphasia for held-out participants at the individual level (accuracy of 83.33%, AUC of 89.16%, see Fig. 4A). The relative contribution of individual frequency bands to classification accuracy further confirmed our group comparison results: delta, theta and gamma band neural tracking were most predictive for capturing aphasia (see Fig. 4B).

As described in the ‘Materials and methods’ section, 8 out of 26 IWA (i.e. 30%) did not score below the cut-off threshold on either of the two diagnostic language tests for aphasia administered during the study.^30,31^ Nonetheless, these patients had an extended language deficit documentation and followed speech-language therapy at the time of participation. Research indicates that standardized behavioural assessments currently used in clinical settings lack sensitivity to detect mild aphasia symptoms, prompting the development of novel tests for mild aphasia (e.g. see Satoer *et al*.^58^). In contrast, the SVM classifier correctly identified 23 out of 26 aphasia cases (two out of three misclassified cases also did not score below the cut-off threshold on either of the two diagnostic language tests). While the higher detection accuracy of the EEG-based neural tracking classification may suggest greater sensitivity compared to standardized behavioural assessment, further investigation, including a validation against behavioural tests for mild aphasia, is warranted. Additionally, attention must be given to enhancing the sensitivity of aphasia detection (77% in the present study), which may involve fine-tuning the classifier to optimize the desired ratio of false to true positive aphasia detection or developing norm scores for neural envelope tracking based on large-sample investigations per age group.

However, comparing neural tracking outcomes to behavioural assessment is difficult, as the underlying language skills tested are different. Standardized aphasia tests use isolated sounds or linguistic units, which do not resemble daily-life, natural speech.^5^ As these tests do not assess natural language abilities, discrepancies exist between test outcomes and a patient's daily-life experiences.^6,59-63^ While there exist behavioural natural speech assessments,^59^ they are not commonly applied in practice due to the high workload (time-intensive, 1 min of naturally produced aphasic speech can take up to 60 min of transcribing and analysing the speech material^64^) and a lack of knowledge of natural speech analyses.^7^ Novel automatic speech recognition and natural language processing techniques^65^ may provide a solution in the future. The neural tracking technique however directly addresses the limitation of low ecological validity from which behavioural tests suffer as it employs natural speech stimuli, and offers a unique perspective as it measures receptive speech processes.

Assessing the reliability and time-efficiency of neural envelope tracking is crucial to evaluate its clinical application potential. However, this was ignored in previous EEG-based studies^66-68^ involving traditional ERP paradigms in aphasia. Therefore, we investigated how much data neural envelope tracking requires to detect aphasia accurately and to yield reliable results. We assessed SVM classification performance as a function of recording time. Our findings indicate that high-accuracy detection can be achieved with just 5 min of recording time (accuracy of 81.25%, see Fig. 5A). Yet, additional minor benefits were acquired with a longer recording duration (AUC robustly crossing 85% from 13 min on). In terms of stability of the metrics, we found that 7 min recording length is sufficient for the TMIF of individual subjects and at the group level to resemble the TMIF from the entire recording (see Fig. 5B and C). These findings were consistent within both groups, with 7 min being the minimum recording length required (as illustrated in the Supplementary Fig. 10). Notably, lower frequency bands (delta and theta) converge faster (3–5 min) and revealed higher correlations compared to higher frequency bands. This can be attributed to a lower signal-to-noise ratio in higher frequency ranges. A minimum recording length of 5–7 min is consistent with previous research in healthy participants (±3–10 min, see Desai *et al*.^69^ and Mesik and Wojtczak^70^).

As for the reliability of neural envelope tracking, we reported high split-half reliability metrics, particularly in the delta and theta bands (ICC > 0.80, see Table 1). Our findings are consistent with previous EEG neural tracking research reporting a correlation of *R* = 0.89 for delta and *R* = 0.82 for theta across two stories in a cohort with language impairments caused by a neurodegenerative disorder.^24^ Although future studies should examine the generalizability of the results across stories and speakers at different test sessions (i.e. test–retest), our results on the required recording time, stability and reliability provide first evidence that neural tracking has the potential to be applied in clinical practice for aphasia screening.

### Limitations and future directions

This study did not target specific subtypes of aphasia or sample patients with particular impairments in speech production or reception; rather, our sample consisted of IWA exhibiting mixed symptoms. Of the 18 IWA failing behavioural language tests, 13 failed both a test for language production (Dutch Naming test) and a test for language reception (Dutch ScreeLing test, see Supplementary Table 1). Over the years, research has consistently shown significant overlap in neural representations for language production and reception, including neural structures that instantiate phonology, semantics and syntax,^71-73^ challenging classical models of aphasia subtypes. Furthermore, research has indicated that over half of IWA cannot be classified into a specific subtype as they present with mixed symptoms, and that substantial heterogeneity exists within aphasia subtypes.^74,75^ Data-driven approaches to clustering aphasia profiles also contrast with traditional subtypes, as they indicate clusters of impaired language components (i.e. phonology, semantics and syntax) rather than a clear distinction between language production and reception.^76^ Together, these findings underscore the inadequacy of classical models in representing the neural substrates of language functions and highlight the need for individualized approaches tailored to specific language processes in each IWA. In this perspective, it is imperative that future research investigates the relationship between neural tracking and the different language components, potentially by relating neural tracking of different frequency bands or higher-order linguistic features^77,78^ to behavioural measures targeting specific language components. While such investigations require a significantly enhanced sample size and more extensive behavioural testing, this paper provides an important first step by demonstrating reduced neural tracking of specific frequency bands (relating to different language processes) in aphasia and the sensitivity of these measures to capture aphasia at the individual level.

In the present study, we did not observe significant associations between lesion size and neural envelope tracking measures in *post hoc* analyses (see Supplementary Table 3). This suggests the specificity of our measures, which are likely not driven by lesion-induced effects. However, future research should delve deeper into the impact of stroke-induced lesions on the obtained neural tracking measures, potentially through methodological comparisons with a non-aphasic stroke group. Such comparisons are often overlooked in EEG research on aphasia,^67,68^ but also in the validation of behavioural tests used in clinical practice, as highlighted by a systematic review.^1^ Nevertheless, these comparisons are essential for assessing the specificity of an assessment tool for post-stroke aphasia. Furthermore, future work should assess neural tracking in IWA in the acute stage after stroke. In this perspective, future work should consider EEG setup and preparation time as well. While our work shows that neural tracking can be estimated in a time-efficient manner, future work could select specific EEG channels (e.g. through utility metrics for channel importance^79^) or consider more practical EEG setups. Finally, our neural tracking approach only considered language stimuli in the receptive domain. Recent work suggests that similar analyses can also be applied to the expressive domain, i.e. speech production,^80^ which could open new perspectives to studying expressive language problems in IWA.

## Conclusion

In conclusion, our findings reveal that neural envelope tracking of natural speech is an effective marker for post-stroke aphasia, capturing language impairments at the individual level in a highly reliable and time-efficient manner. Although future investigations are warranted prior to clinical implementations, including validation against a non-aphasic stroke group and assessment of the technique's sensitivity to capture specific impaired language processes, our work represents an important first step towards more automatic and ecologically valid assessments of language problems in aphasia.

## Supplementary Material

fcaf095_Supplementary_Data

## Data Availability

We shared our neural tracking outcomes (i.e. the TMIFs) on the Open Science Framework: https://osf.io/nkmfa/. Note that our ethical approval does not permit public archiving of raw neuroimaging data, but raw EEG data can be made available upon request and if the GDPR-related conditions are met.

## References

[1] Rohde A, Worrall L, Godecke E, O’Halloran R, Farrell A, Massey M. Diagnosis of aphasia in stroke populations: A systematic review of language tests. PLoS One. 2018;13(3):e0194143. 29566043 10.1371/journal.pone.0194143 PMC5863973

[2] Aerts A, Mierlo P, Hartsuiker RJ, Santens P, Letter MD. Neurophysiological sensitivity for impaired phonological processing in the acute stage of aphasia. Brain Lang. 2015;149:84–96. 26197257 10.1016/j.bandl.2015.07.001

[3] Robson H, Pilkington E, Evans L, DeLuca V, Keidel JL. Phonological and semantic processing during comprehension in Wernicke’s aphasia: An N400 and Phonological Mapping Negativity study. Neuropsychologia. 2017;100:144–154. 28433347 10.1016/j.neuropsychologia.2017.04.012

[4] Cocquyt EM, Vandewiele M, Bonnarens C, Santens P, De Letter M. The sensitivity of event-related potentials/fields to logopedic interventions in patients with stroke-related aphasia. Acta Neurol Belg. 2020;120(4):805–817. 32474880 10.1007/s13760-020-01378-3

[5] Hamilton LS, Huth AG. The revolution will not be controlled: Natural stimuli in speech neuroscience. Lang Cogn Neurosci. 2018;35(5):573–582. 32656294 10.1080/23273798.2018.1499946 PMC7324135

[6] Lesser R, Algar L. Towards combining the cognitive neuropsychological and the pragmatic in aphasia therapy. Neuropsychol Rehabil. 1995;5(1–2):67–92.

[7] Stark BC, Dutta M, Murray LL, et al Spoken discourse assessment and analysis in aphasia: An international survey of current practices. J Speech Lang Hear Res. 2021;64(11):4366–4389. 34554878 10.1044/2021_JSLHR-20-00708 PMC9132151

[8] Peelle JE, Davis MH. Neural oscillations carry speech rhythm through to comprehension. Front Psychol. 2012;3:320. 22973251 10.3389/fpsyg.2012.00320 PMC3434440

[9] Shannon RV, Zeng FG VK, Wygonski J SEM. Speech recognition with primarily temporal cues. Science. 1995;270(5234):303–304. 7569981 10.1126/science.270.5234.303

[10] Crosse MJ, Zuk NJ, Di Liberto GM, Nidiffer AR, Molholm S, Lalor EC. Linear modeling of neurophysiological responses to speech and other continuous stimuli: Methodological considerations for applied research. Front Neurosci. 2021;15:705621. 34880719 10.3389/fnins.2021.705621 PMC8648261

[11] De Clercq P, Vanthornhout J, Vandermosten M, Francart T. Beyond linear neural envelope tracking: A mutual information approach. J Neural Eng. 2023;20(2):026007. 10.1088/1741-2552/acbe1d 36812597

[12] Brodbeck C, Das P, Gillis M, et al Eelbrain: A Python toolkit for time-continuous analysis with temporal response functions. Elife. 2023:12:e85012 38018501 10.7554/eLife.85012 PMC10783870

[13] Ding N, Simon JZ. Adaptive temporal encoding leads to a background-insensitive cortical representation of speech. J Neurosci. 2013;33(13):5728–5735. 23536086 10.1523/JNEUROSCI.5297-12.2013 PMC3643795

[14] Etard O, Reichenbach T. Neural speech tracking in the theta and in the delta frequency band differentially encode clarity and comprehension of speech in noise. J Neurosci. 2019;39(29):5750–5759. 31109963 10.1523/JNEUROSCI.1828-18.2019 PMC6636082

[15] Vanthornhout J, Decruy L, Wouters J, Simon JZ, Francart T. Speech intelligibility predicted from neural entrainment of the speech envelope. JARO—J Assoc Res Otolaryngol. 2018;19(2):181–191. 10.1007/s10162-018-0654-z PMC5878153 29464412

[16] Gillis M, Van Canneyt J, Francart T, Vanthornhout J. Neural tracking as a diagnostic tool to assess the auditory pathway. Hear Res. 2022;426:108607. 36137861 10.1016/j.heares.2022.108607

[17] Giraud A, Poeppel D. Cortical oscillations and speech processing: Emerging computational principles and operations. Nat Neurosci. 2012;15:511–517. 22426255 10.1038/nn.3063 PMC4461038

[18] Ding N, Melloni L, Zhang H, Tian X, Poeppel D. Cortical tracking of hierarchical linguistic structures in connected speech. Nat Neurosci. 2016;19:158–164. 26642090 10.1038/nn.4186 PMC4809195

[19] Kaufeld G, Bosker HR, Alday PM, Meyer AS, Martin AE. Linguistic structure and meaning organize neural oscillations into a content-specific hierarchy. J Neurosci. 2020;40(49):9467–9475. 33097640 10.1523/JNEUROSCI.0302-20.2020 PMC7724143

[20] Lizarazu M, Lallier M, Molinaro N. Phase amplitude coupling between theta and gamma oscillations adapts to speech rate. Ann N Y Acad Sci. 2019;1453(1):140–152. 31020680 10.1111/nyas.14099 PMC6850406

[21] Wöstmann M, Lim S, Obleser J. The human neural alpha response to speech is a proxy of attentional control. Cereb Cortex. 2017;27(6):3307–3317. 28334352 10.1093/cercor/bhx074

[22] Fujioka T, Ross B, Trainor LJ. Beta-band oscillations represent auditory beat and its metrical hierarchy in perception and imagery. J Neurosci. 2015;35(45):15187–15198. 26558788 10.1523/JNEUROSCI.2397-15.2015 PMC6605356

[23] Hyafil A, Giraud AL, Fontolan L, Gutkin B. Neural cross-frequency coupling: Connecting architectures, mechanisms, and functions. Trends Neurosci. 2015;38(11):725–740. 26549886 10.1016/j.tins.2015.09.001

[24] Dial HR, Gnanateja GN, Tessmer RS, Gorno-Tempini ML, Chandrasekaran B, Henry ML. Cortical tracking of the speech envelope in logopenic variant primary progressive aphasia. Front Hum Neurosci. 2021;14:597694. 33488371 10.3389/fnhum.2020.597694 PMC7815818

[25] Di Liberto GM, Lalor EC. Indexing cortical entrainment to natural speech at the phonemic level: Methodological considerations for applied research. Hear Res. 2017;348:70–77. 28246030 10.1016/j.heares.2017.02.015

[26] Lizarazu M, Lallier M, Bourguignon M, Carreiras M, Molinaro N. Impaired neural response to speech edges in dyslexia. Cortex. 2021;135:207–218. 33387899 10.1016/j.cortex.2020.09.033

[27] Mandke K, Flanagan S, Macfarlane A, et al Neural sampling of the speech signal at different timescales by children with dyslexia. NeuroImage. 2022;253:119077. 35278708 10.1016/j.neuroimage.2022.119077

[28] Flamand-Roze C, Falissard B, Roze E, et al Validation of a new language screening tool for patients with acute stroke: The Language Screening Test (LAST). Stroke. 2011;42(5):1224–1229. 21487118 10.1161/STROKEAHA.110.609503

[29] Kries J, De Clercq P, Lemmens R, Francart T, Vandermosten M. Acoustic and phonemic processing are impaired in individuals with aphasia. Sci Rep. 2023;13(11208):1–15. 37433805 10.1038/s41598-023-37624-w PMC10336064

[30] van Ewijk L, Dijkhuis L, Hofs-van Kats M, Hendrickx-Jessurun M, Wijngaarden M, de Hilster C. Handleiding Nederlandse Benoem Test. 2nd ed. Houten: Bohn Stafleu van Loghum; 2020.

[31] El Hachioui H, Visch-Brink EG, Lau LM, et al Screening tests for aphasia in patients with stroke: A systematic review. J Neurol. 2017;264(2):211–220. doi:10.1007/s00415-016-8170-8 27260296 PMC5306063

[32] Visch-Brink E, Van de Sandt-Koenderman M, El Hachioui H. ScreeLing. Houten: Bohn Stafleu van Loghum; 2010.

[33] Schevenels K, Michiels L, Lemmens R, De Smedt B, Zink I, Vandermosten M. The role of the hippocampus in statistical learning and language recovery in persons with post stroke aphasia. Neuroimage Clin. 2022;36:103243. 36306718 10.1016/j.nicl.2022.103243 PMC9668653

[34] Jansen S, Luts H, Wagener KC, et al Comparison of three types of French speech-in-noise tests: A multi-center study. Int J Audiol. 2012;51(3):164–173. 22122354 10.3109/14992027.2011.633568

[35] Søndergaard P, Torrésani B, Balazs P. The linear time frequency analysis toolbox. Int J Wavelets Multiresolut Inf Process. 2012;10:1250032.

[36] Pedroni A, Bahreini A, Langer N. Automagic: Standardized preprocessing of big EEG data. NeuroImage. 2019;200:460–473. 31233907 10.1016/j.neuroimage.2019.06.046

[37] de Cheveigné A . ZapLine: A simple and effective method to remove power line artifacts. NeuroImage. 2020;207:116356. 31786167 10.1016/j.neuroimage.2019.116356

[38] Bigdely-Shamlo N, Mullen T, Kothe C, Su KM, Robbins KA. The PREP pipeline: Standardized preprocessing for large-scale EEG analysis. Front Neuroinform. 2015;9:16. 26150785 10.3389/fninf.2015.00016 PMC4471356

[39] Mullen TR, Kothe CAE, Chi YM, et al Real-time neuroimaging and cognitive monitoring using wearable dry EEG. IEEE Trans Biomed Eng. 2015;11(62):2553–2567. 10.1109/TBME.2015.2481482 PMC4710679 26415149

[40] Pion-Tonachini L, Kreutz-Delgado K, Makeig S. ICLabel: An automated electroencephalographic independent component classifier, dataset, and website. NeuroImage. 2019;198:181–197. 31103785 10.1016/j.neuroimage.2019.05.026 PMC6592775

[41] Ince RAA, Giordano B, Kayser C, Rousselet G, Gross J, Schyns P. A statistical framework for neuroimaging data analysis based on mutual information estimated via a Gaussian copula. Hum Brain Mapp. 2017;38(3):1541–1573. 27860095 10.1002/hbm.23471 PMC5324576

[42] Zan P, Presacco A, Anderson S, Simon JZ. Exaggerated cortical representation of speech in older listeners: Mutual information analysis. J Neurophysiol. 2020;124(4):1152–1164. 32877288 10.1152/jn.00002.2020 PMC7717162

[43] Van Hirtum T, Somers B, Dieudonné B, et al Neural envelope tracking predicts speech intelligibility and hearing aid benefit in children with hearing loss. Hear Res. 2023;439:108893. 37806102 10.1016/j.heares.2023.108893

[44] Lesenfants D, Vanthornhout J, Verschueren E, Decruy L, Francart T. Predicting individual speech intelligibility from the neural tracking of acoustic- and phonetic-level speech representations. Hear Res. 2019;380:1–9. 31167150 10.1016/j.heares.2019.05.006

[45] Maris E, Oostenveld R. Nonparametric statistical testing of EEG- and MEG-data. J Neurosci Methods. 2007;164(1):177–190. 17517438 10.1016/j.jneumeth.2007.03.024

[46] Pedregosa F, Varoquaux G, Gramfort A, et al Scikit-Learn: Machine learning in Python. J Mach Learn Res. 2011;12:2825–2830.

[47] Decruy L, Vanthornhout J, Francart T. Evidence for enhanced neural tracking of the speech envelope underlying age-related speech-in-noise difficulties. J Neurophysiol. 2019;122(2):601–615. 31141449 10.1152/jn.00687.2018 PMC6734401

[48] Lundberg SM, Lee SI. A unified approach to interpreting model predictions. Adv Neural Inf Process. 2017;30:6785–6795.

[49] Satopaa V, Albrecht J, Irwin D, Raghavan B. Finding a “Kneedle” in a haystack: detecting knee points in system behavior. In: *2011 31st International Conference on Distributed Computing Systems Workshops*, *Minneapolis, MN, USA*. 2011:166-171. doi:10.1109/ICDCSW.2011.20

[50] Chen YP, Schmidt F, Keitel A, Rösch S, Hauswald A, Weisz N. Speech intelligibility changes the temporal evolution of neural speech tracking. NeuroImage. 2023;268:119894. 36693596 10.1016/j.neuroimage.2023.119894

[51] Verschueren E, Gillis M, Decruy L, Vanthornhout J, Francart T. Speech understanding oppositely affects acoustic and linguistic neural tracking in a speech rate manipulation paradigm. J Neurosci. 2022;42(39):7442–7453. 36041851 10.1523/JNEUROSCI.0259-22.2022 PMC9525161

[52] Keitel A, Gross J, Kayser C. Perceptually relevant speech tracking in auditory and motor cortex reflects distinct linguistic features. PLoS Biol. 2018;16(3):e2004473. 29529019 10.1371/journal.pbio.2004473 PMC5864086

[53] Coopmans CW, de Hoop H, Hagoort P, Martin AE. Effects of structure and meaning on cortical tracking of linguistic units in naturalistic speech. Neurobiol Lang. 2022;3(3):386–412. 10.1162/nol_a_00070 PMC10158633 37216060

[54] Leonard M, Baud M, Sjerp M, et al Perceptual restoration of masked speech in human cortex. Nat Commun. 2016;7:13619. 27996973 10.1038/ncomms13619 PMC5187421

[55] Di Liberto GM, Lalor EC, Millman RE. Causal cortical dynamics of a predictive enhancement of speech intelligibility. NeuroImage. 2018;166:247–258. 29102808 10.1016/j.neuroimage.2017.10.066

[56] Xu N, Zhao B, Luo L, et al Two stages of speech envelope tracking in human auditory cortex modulated by speech intelligibility. Cereb Cort. 2022;33(5):2215–2228. 10.1093/cercor/bhac203 35695785

[57] Gross J, Hoogenboom N, Thut G, et al Speech rhythms and multiplexed oscillatory sensory coding in the human brain. PLoS Biol. 2013;11(12):e1001752. 24391472 10.1371/journal.pbio.1001752 PMC3876971

[58] Satoer D, De Witte E, Bulté B, et al Dutch Diagnostic Instrument for Mild Aphasia (DIMA): Standardisation and a first clinical application in two brain tumour patients. Clin Linguist Phon. 2022;36(11):929–953. 35899484 10.1080/02699206.2021.1992797

[59] Armstrong E . Aphasic discourse analysis: The story so far. Aphasiology. 2000;14(9):875–892.

[60] Wallace SE, Kimelman MD. Generalization of word retrieval following semantic feature treatment. NeuroRehabilitation. 2013;32(4):899–913. 23867416 10.3233/NRE-130914

[61] Stark BC, Fukuyama J. Leveraging big data to understand the interaction of task and language during monologic spoken discourse in speakers with and without aphasia. Lang Cogn Neurosci. 2021;36(5):562–585.

[62] Kim H, Berube S, Hillis AE. Core lexicon in aphasia: A longitudinal study. Aphasialogy. 2022;37(10):1679–1691. 10.1080/02687038.2022.2121598 PMC10564387 37822874

[63] Hickin J, Cruice M, Dipper L. A feasibility study of a nove computer-based treatment for sentence production deficits in aphasia, delivered by a combination of clinician-led and self-managed treatment sessions. Aphasialogy. 2022;37(10):1623–1645.

[64] Armstrong L, Brady M, Mackenzie C, Norrie J. Transcription-less analysis of aphasia discourse: A clinician’s dream or a possibility? Aphasialogy. 2007;21(3–4):355–374.

[65] Dalton SG, Stark BC, Fromm D, et al Validation of an automated procedure for calculating core lexicon from transcripts. J Speech Lang Hear Res. 2022;65(8):2996–3003. 35917459 10.1044/2022_JSLHR-21-00473 PMC9911121

[66] Meechan RJ, McCann CM, Purdy SC. The electrophysiology of aphasia: A scoping review. Clin Neurophysiol. 2021;132(12):3025–3034. 34717223 10.1016/j.clinph.2021.08.023

[67] Silkes JAP, Anjum J. The role and use of event-related potentials in aphasia: A scoping review. Brain Lang. 2021;219:104966. 34044294 10.1016/j.bandl.2021.104966

[68] Cocquyt EM, Van Laeken H, van Mierlo P, De Letter M. Test–retest reliability of electroencephalographic and magnetoencephalographic measures elicited during language tasks: A literature review. Eur J Neurosci. 2023;57(8):1353–1367. 36864752 10.1111/ejn.15948

[69] Desai M, Field AM, Hamilton LS. Dataset size considerations for robust acoustic and phonetic speech encoding models in EEG. Front Hum Neurosci. 2023;16:1001171. 36741776 10.3389/fnhum.2022.1001171 PMC9895838

[70] Mesik J, Wojtczak M. The effects of data quantity on performance of temporal response function analyses of natural speech processing. Front Neurosci. 2023:16:963629. 36711133 10.3389/fnins.2022.963629 PMC9878558

[71] Tremblay P, Dick AS. Broca and Wernicke are dead, or moving past the classic model of language neurobiology. Brain Lang. 2016;162:60–71. 27584714 10.1016/j.bandl.2016.08.004

[72] Hickok G, Poeppel D. The cortical organization of speech processing. Nat Rev Neurosci. 2007;8(5):393–402. 17431404 10.1038/nrn2113

[73] Fedorenko E, Ivanova AA, Regev TI. The language network as a natural kind within the broader landscape of the human brain. Nat Rev Neurosci. 2024;25(5):289–312. 38609551 10.1038/s41583-024-00802-4 PMC13222024

[74] Bastiaanse R . Afasie. *Bohn Stafleu van Loghum*. Houten: Bohn Stafleu van Loghum; 2011.

[75] Kemmerer D . Cognitive neuroscience of language. Psychology Press (Taylor & Francis); 2015.

[76] Landrigan JF, Zhang F, Mirman D. A data-driven approach to post-stroke aphasia classification and lesion-based prediction. Brain. 2021;144(5):1372–1383. 34046670 10.1093/brain/awab010 PMC8219353

[77] Gillis M, Vanthornhout J, Simon JZ, Francart T, Brodbeck C. Neural markers of speech comprehension: Measuring EEG tracking of linguistic speech representations, controlling the speech acoustics. J Neurosci. 2021;41(50):10316–10329. 34732519 10.1523/JNEUROSCI.0812-21.2021 PMC8672699

[78] Gillis M, Kries J, Vandermosten M, Francart T. Neural tracking of linguistic and acoustic speech representations decreases with advancing age. NeuroImage. 2023;267(119841):1–16. 10.1016/j.neuroimage.2022.119841 PMC9878439 36584758

[79] Montoya-Martínez J, Vanthornhout J, Bertrand A, Francart T. Effect of number and placement of EEG electrodes on measurement of neural tracking of speech. PLoS One. 2021;16(2):e0246769. 33571299 10.1371/journal.pone.0246769 PMC7877609

[80] Perez A, Davis MH, Ince RAA, et al Timing of brain entrainment to the speech envelope during speaking, listening and self-listening. Cognition. 2022;224:105051. 35219954 10.1016/j.cognition.2022.105051 PMC9112165

