# Supplementary material for: Neural tracking of natural speech: an effective marker for post-stroke aphasia
Source: Brain Commun. 2025 Mar 10;7(2):fcaf095. doi: 10.1093/braincomms/fcaf095 (PMC11891514; doi:10.1093/braincomms/fcaf095)
Supplement: fcaf095_Supplementary_Data [file fcaf095_supplementary_data.zip › Supplementary_material.pdf]

# Supplementary Materials

## Responses to content questions of the presented story

Participants listened to the presented story in blocks of approximately 5 minutes, and responded to content questions in between the different blocks. This included both yes/no questions and multiple choice questions. IWA scored significantly lower on both types of questions (yes/no: W=149, p=0.003; multiple choice: W=145, p=0.003; Wilcoxon rank-sum tests). As these questions were not validated, this result should not be interpreted, but instead be seen as descriptive. Results are visualized through boxplots in Supplementary Figure 1.

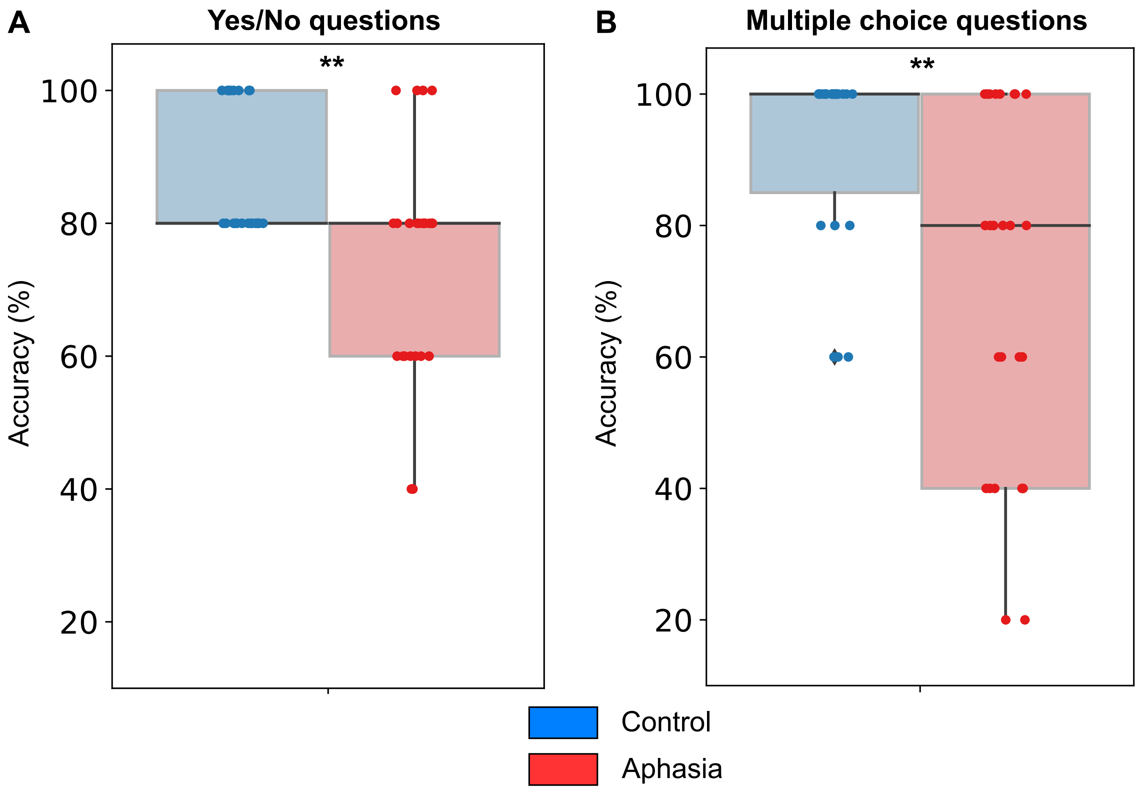


**Supplementary Figure 1. Responses to content questions**. **A.** Accuracy of responses to yes/no questions. **B.** Accuracy of responses to multiple-choice questions. **= p<.01.

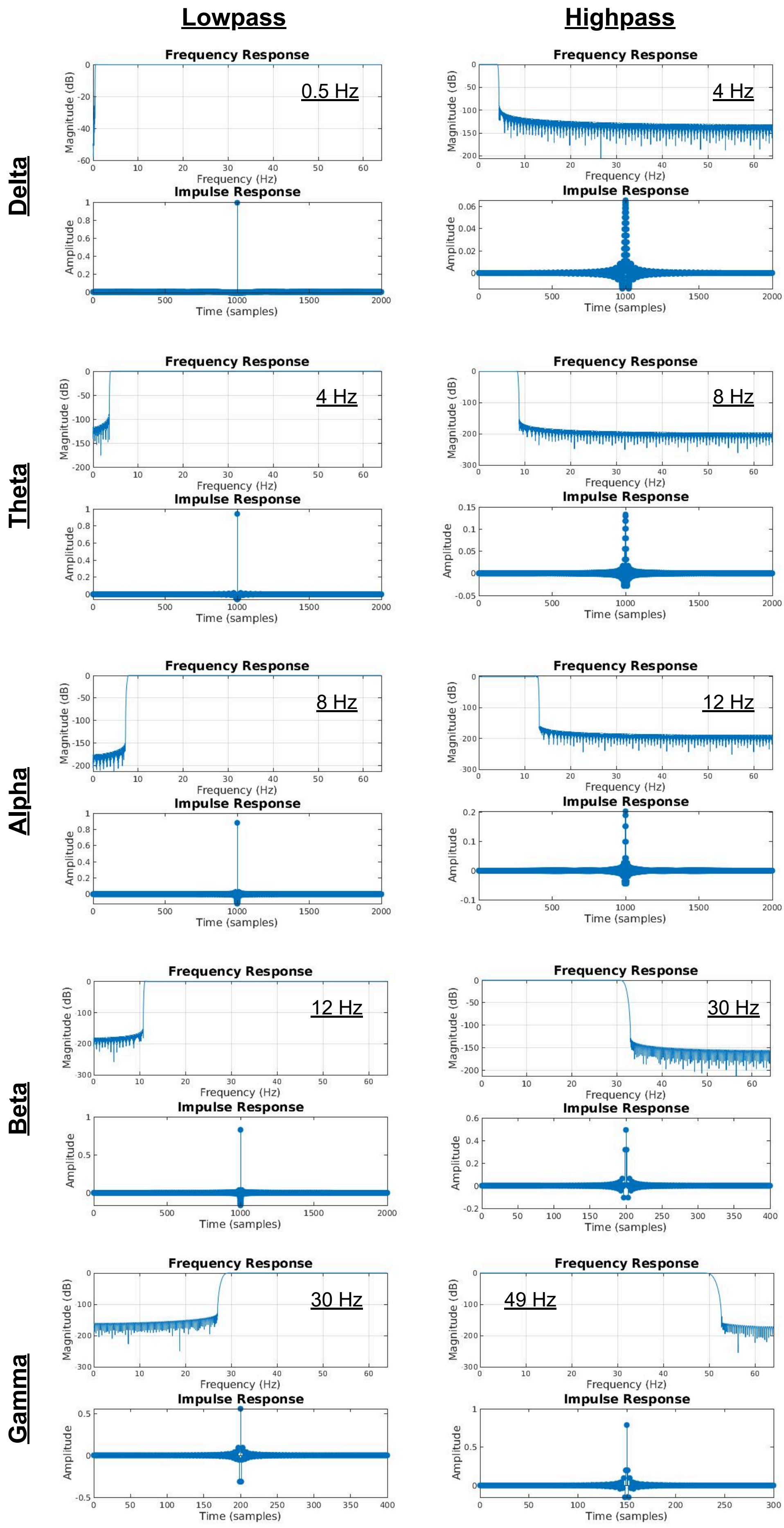


**Supplementary Figure 2. Impulse and frequency response of all applied filters**. For the broadband frequency range, we applied a lowpass filter of 0.5 Hz (i.e., same lowpass filter of delta band) and a highpass filter of 49 Hz (same highpass filter of gamma band).

## Channel Selection

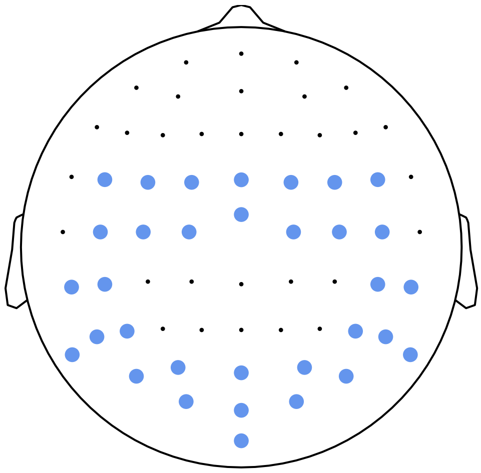

**Supplementary Figure 3. Channel selection.**

## Significance level of neural envelope tracking

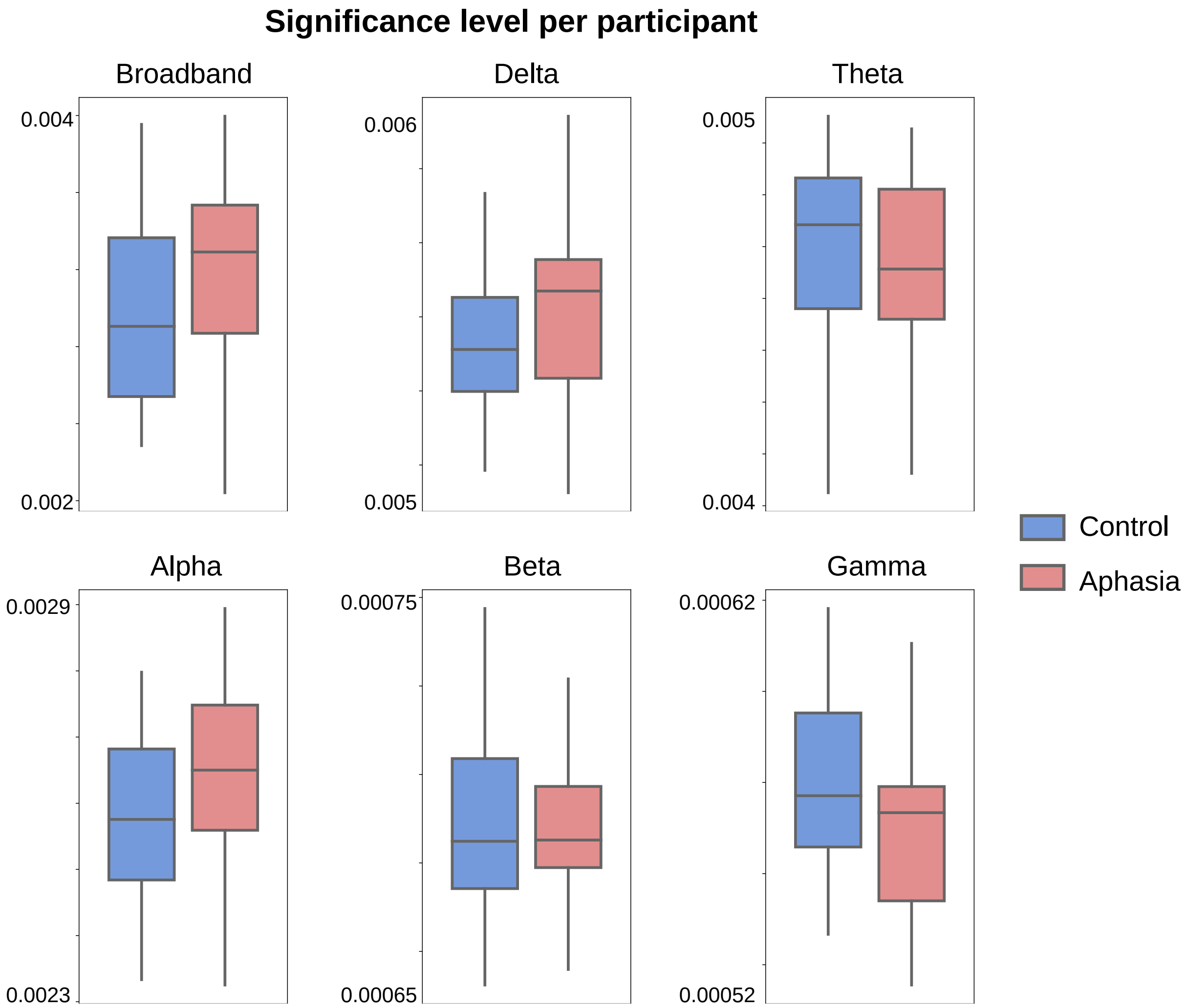


**Supplementary Figure 4. Significance level of neural tracking.** Boxes represent the 95th percentile of permutations per subject and per frequency band. There was no significant difference between groups for any frequency band.

## Single-channel TMIF analysis

### Delta band

The single-channel TMIF analysis revealed decreased delta band envelope tracking for IWA compared to healthy controls. A spatio-temporal cluster-based permutation test identified a cluster (p=0.008) comprising a large group of bilateral fronto-central, parietal and posterior channels (N = 39 channels) and brain latencies from 0.10 s to 0.32 s. The results are depicted in Supplementary Figure 5.

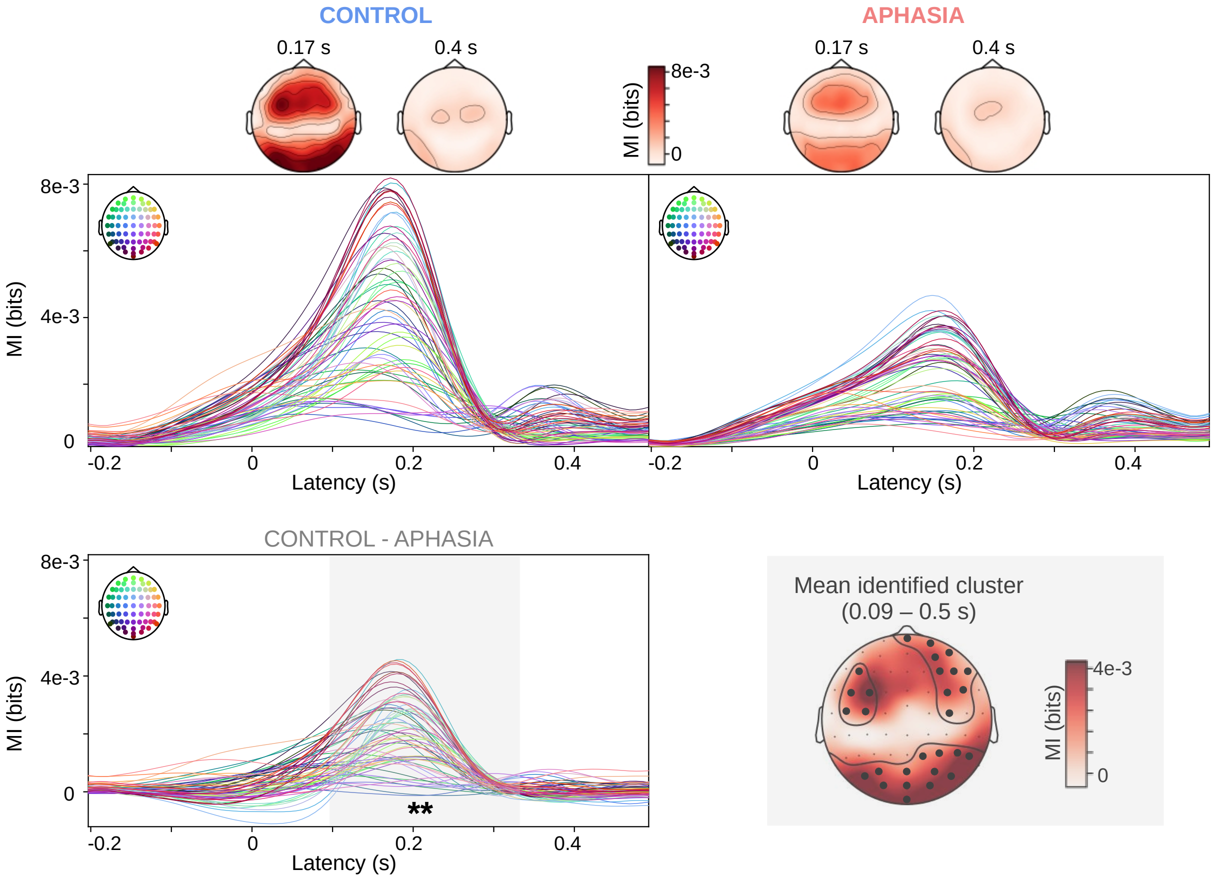


**Supplementary Figure 5. Delta band analysis.** The average single-channel TMIF in delta band for the control and the aphasia group separately, with topoplots at indicated brain latencies. The spatio-temporal cluster-based permutation test investigated the difference between the control and aphasia group (control - aphasia) and identified a cluster (below threshold p<0.05) with the largest group difference. Brain latencies belonging to the cluster are marked in a shaded gray area, the channels belonging to the cluster are indicated with a black dot on the topoplot.** = p<0.01

## Theta band

For the theta band, the single-channel TMIF analysis revealed decreased envelope tracking for IWA compared to healthy controls. A spatio-temporal cluster-based permutation test identified a cluster ($p<0.001$) comprising a large group of bilateral fronto-central, parietal and posterior channels (N = 40 channels) and brain latencies from 0.01 s to 0.5 s. Supplementary Figure 6 visualizes the result.

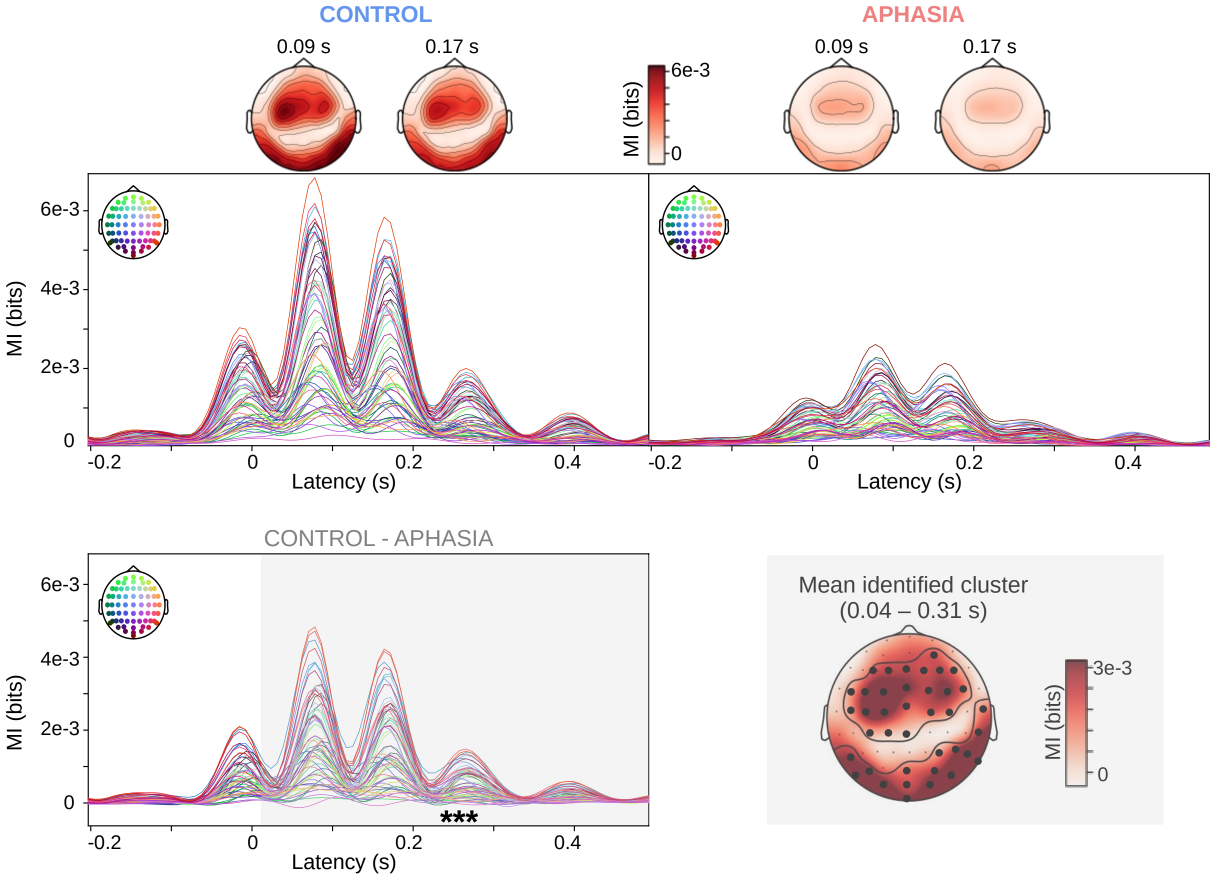


**Supplementary Figure 6. Theta band analysis.** The average single-channel TMIF in theta band for the control and the aphasia group separately, with topoplots at indicated brain latencies. The spatio-temporal cluster-based permutation test investigated the difference between the control and aphasia group (control - aphasia) and identified a cluster (below threshold $p<0.05$) with the largest group difference. Brain latencies belonging to the cluster are marked in a shaded gray area, the channels belonging to the cluster are indicated with a black dot on the topoplot.** = $p<0.01$

**Alpha band**

In the alpha band, a spatio-temporal cluster-based permutation test found no clusters exceeding p<0.05 threshold level. The group results are displayed in Supplementary Figure 7.

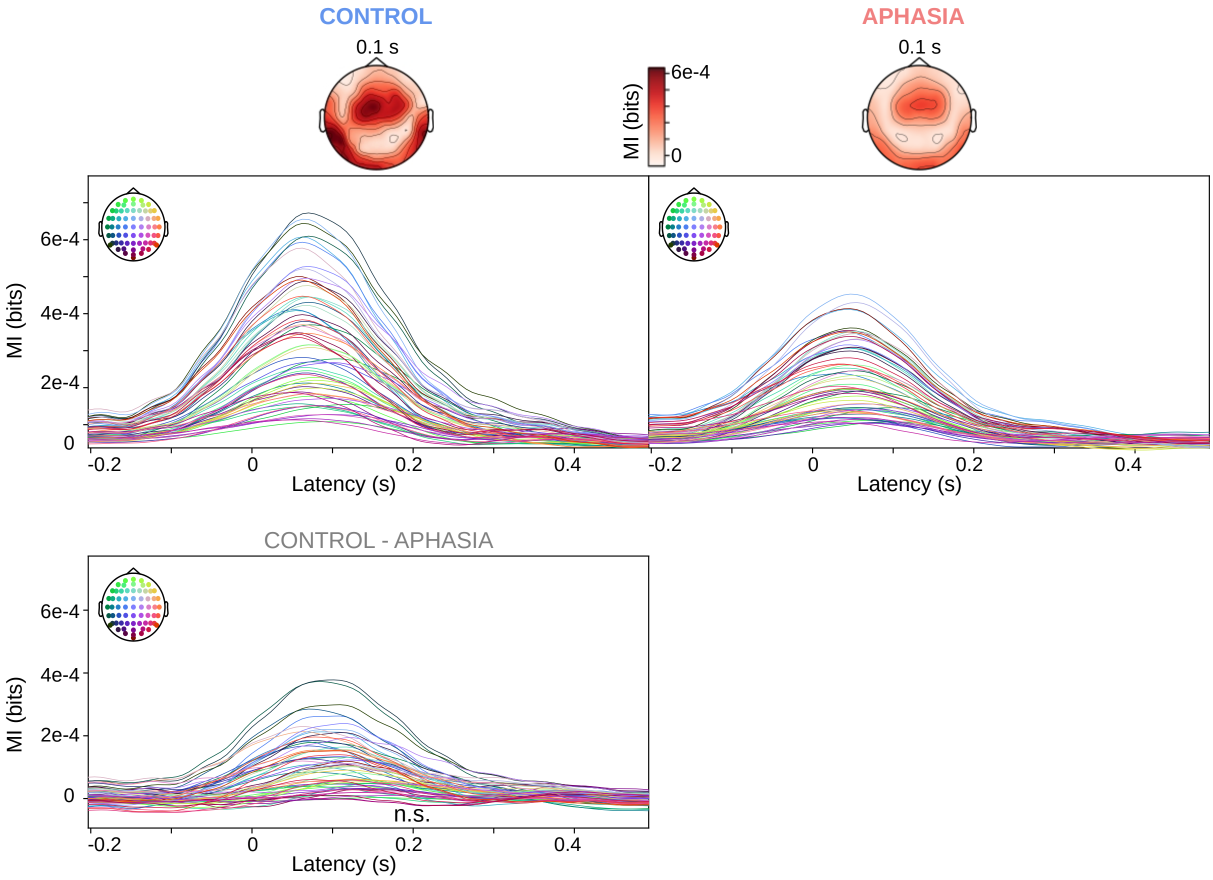


**Supplementary Figure 7. Alpha band analysis.** The average single-channel TMIF in alpha band for the control and the aphasia group separately, with topoplots at indicated brain latencies. The spatio-temporal cluster-based permutation test investigated the difference between the control and aphasia group (control - aphasia), but did not find a group difference with p-value below threshold level 0.05.

## Beta band

In the beta band, a spatio-temporal cluster-based permutation test found no clusters exceeding p<0.05 threshold level. The group results are displayed in Supplementary Figure 8.

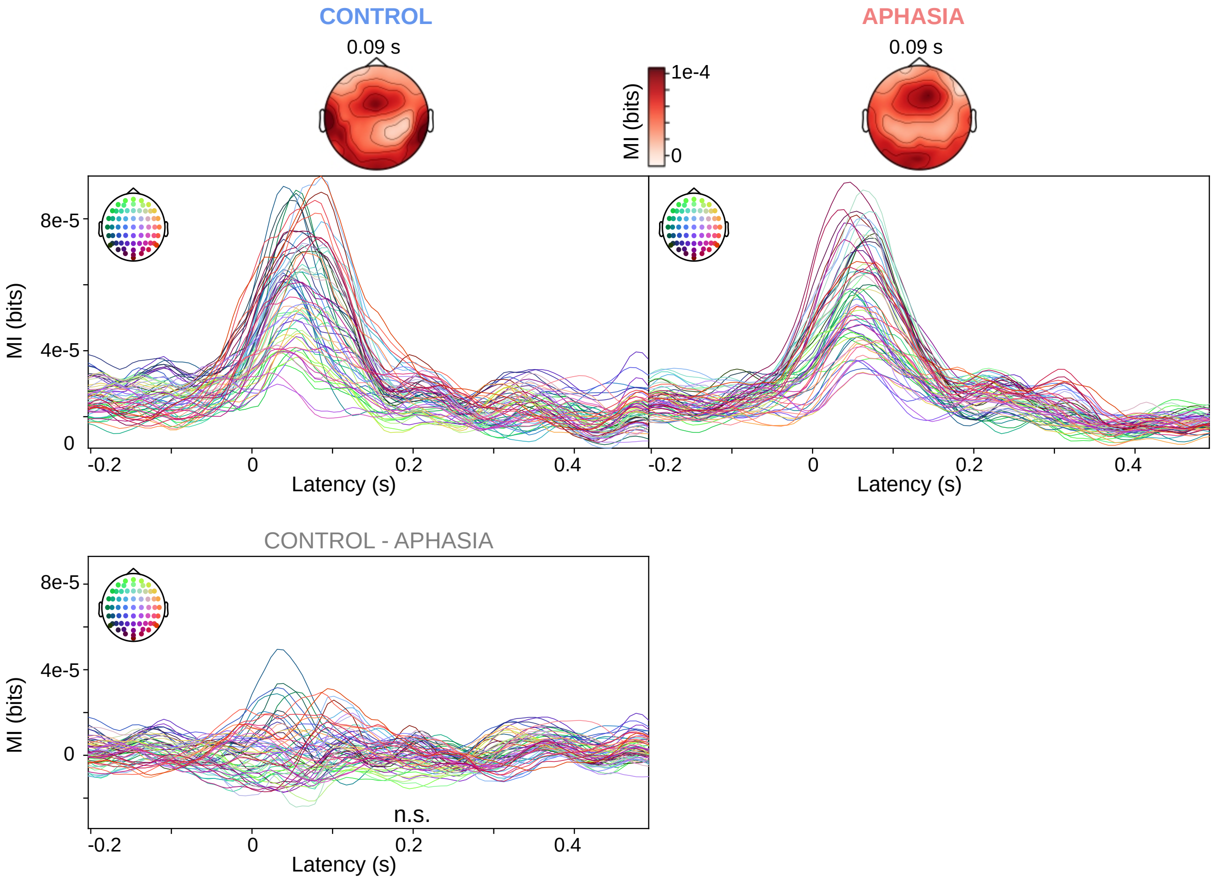


**Supplementary Figure 8. Beta band analysis.** The average single-channel TMIF in beta band for the control and the aphasia group separately, with topoplots at indicated brain latencies. The spatio-temporal cluster-based permutation test investigated the difference between the control and aphasia group (control - aphasia), but did not find a group difference with p-value below threshold level 0.05.

## Gamma band

Finally, IWA displayed decreased neural envelope tracking in the gamma band. A spatio-temporal cluster-based permutation test identified a cluster (p=0.03) comprising parietal and posterior channels (N = 14 channels), primarily in the right hemisphere, and brain latencies from 0.01 s to 0.10 s. Supplementary Figure 9 visualizes the result.

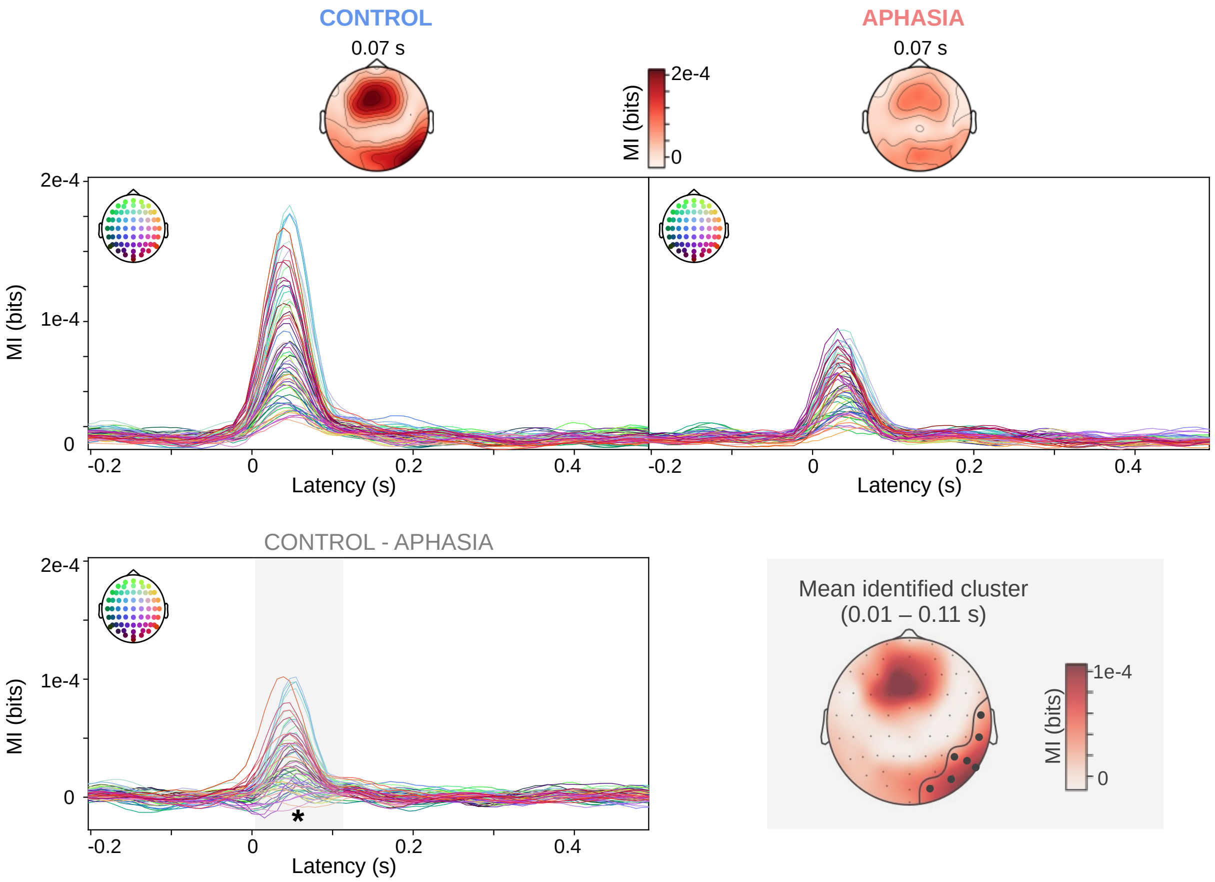


**Supplementary Figure 9. Gamma band analysis.** The average single-channel TMIF in gamma band for the control and the aphasia group separately, with topoplots at indicated brain latencies. The spatio-temporal cluster-based permutation test investigated the difference between the control and aphasia group (control - aphasia) and identified a cluster (below threshold p<0.05) with the largest group difference. Brain latencies belonging to the cluster are marked in a shaded gray area, the channels belonging to the cluster are indicated with a black dot on the topoplot.* = p<0.05

## Group-specific stability analysis

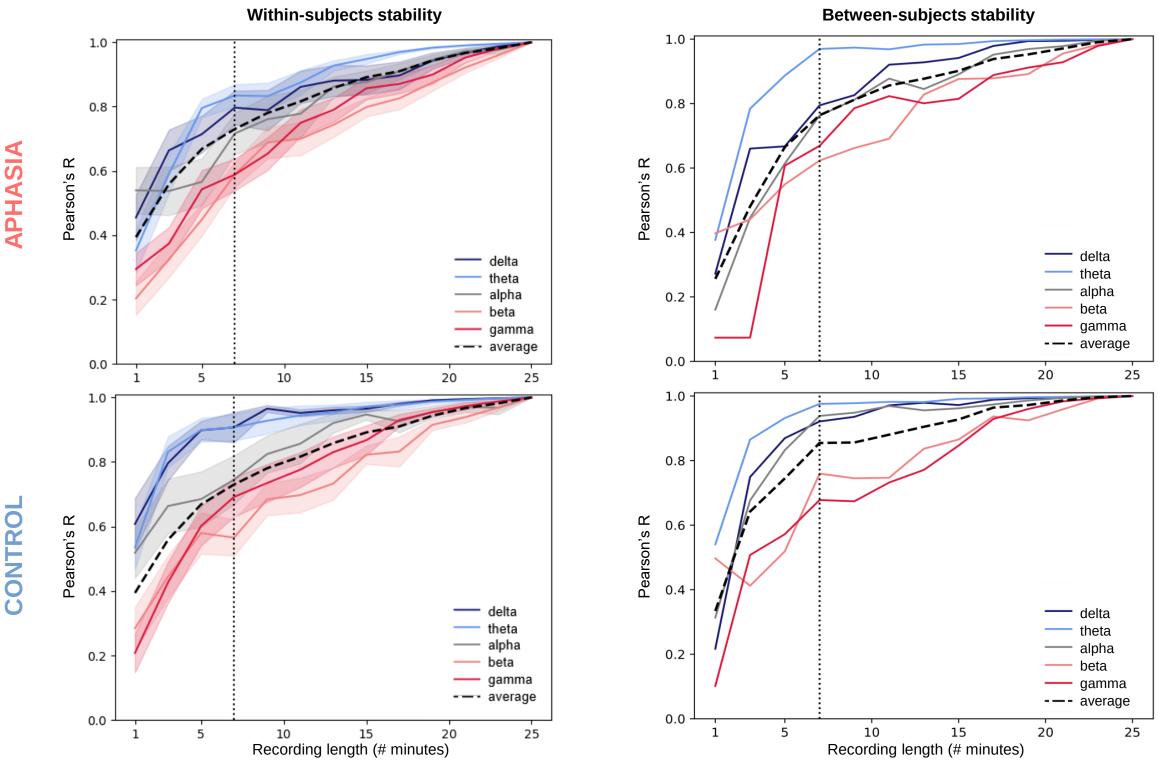


**Supplementary Figure 10. Stability measures grouped**. Within- and between-subjects stability analysis performed for each group separately. Black dotted line indicates the average across frequencies. Shaded areas indicate the standard error of the correlations. The knee point of all panels is indicated with a vertical dotted line (based on the average across frequencies)

**Supplementary Table 2.** Correlation matrix neural envelope tracking

| | IWA | | | | | | Controls | | | | | |
|---|---|---|---|---|---|---|---|---|---|---|---|---|
| | broad | delta | theta | alpha | beta | gamma | broad | delta | theta | alpha | beta | gamma |
| broad | 1 | | | | | | 1 | | | | | |
| delta | 0.79 | 1 | | | | | 0.92 | 1 | | | | |
| theta | 0.15 | 0.01 | 1 | | | | 0.09 | 0.02 | 1 | | | |
| alpha | 0.15 | 0.07 | 0.71 | 1 | | | 0.27 | 0.15 | 0.52 | 1 | | |
| beta | 0.15 | 0.20 | 0.62 | 0.61 | 1 | | 0.48 | 0.57 | 0.32 | 0.44 | 1 | |
| gamma | 0.19 | 0.22 | -0.01 | 0.01 | 0.23 | 1 | 0.12 | 0.18 | -0.18 | -0.22 | 0.26 | 1 |

Exploratory analysis investigating the collinearity between frequency bands. The table displays the Pearson correlations for the mean MI (integration window 0-400 ms).

**Supplementary Table 3.** Correlations stroke size and neural envelope tracking

| | **Lesion size** |
|---|---|
| **broad** | -0.17 (p=0.43) |
| **delta** | -0.24 (p=0.25) |
| **theta** | -0.07 (p=0.74) |
| **alpha** | 0.01 (p=0.98) |
| **beta** | 0.21 (p=0.30) |
| **gamma** | -0.08 (p=0.71) |

Exploratory analysis investigating the association between the lesioned tissue size and neural envelope tracking. The table displays the Pearson correlations for the mean (multivariate) MI (integration window 0-400 ms).
